# A Meta-Analytic Review of Stand-Alone Interventions to Improve Body Image

**DOI:** 10.1371/journal.pone.0139177

**Published:** 2015-09-29

**Authors:** Jessica M. Alleva, Paschal Sheeran, Thomas L. Webb, Carolien Martijn, Eleanor Miles

**Affiliations:** 1 Department of Clinical Psychological Science, Maastricht University, Maastricht, the Netherlands; 2 Department of Psychology and Neuroscience, University of North Carolina at Chapel Hill, Chapel Hill, North Carolina, United States of America; 3 Department of Psychology, University of Sheffield, Sheffield, England; 4 School of Psychology, University of Sussex, Brighton, England; Newcastle University, UNITED KINGDOM

## Abstract

**Objective:**

Numerous stand-alone interventions to improve body image have been developed. The present review used meta-analysis to estimate the effectiveness of such interventions, and to identify the *specific change techniques* that lead to improvement in body image.

**Methods:**

The inclusion criteria were that (a) the intervention was stand-alone (i.e., solely focused on improving body image), (b) a control group was used, (c) participants were randomly assigned to conditions, and (d) at least one pretest and one posttest measure of body image was taken. Effect sizes were meta-analysed and moderator analyses were conducted. A taxonomy of 48 change techniques used in interventions targeted at body image was developed; all interventions were coded using this taxonomy.

**Results:**

The literature search identified 62 tests of interventions (*N* = 3,846). Interventions produced a small-to-medium improvement in body image (*d*_+_ = 0.38), a small-to-medium reduction in beauty ideal internalisation (*d*_+_ = -0.37), and a large reduction in social comparison tendencies (*d*_+_ = -0.72). However, the effect size for body image was inflated by bias both within and across studies, and was reliable but of small magnitude once corrections for bias were applied. Effect sizes for the other outcomes were no longer reliable once corrections for bias were applied. Several features of the sample, intervention, and methodology moderated intervention effects. Twelve change techniques were associated with improvements in body image, and three techniques were contra-indicated.

**Conclusions:**

The findings show that interventions engender only small improvements in body image, and underline the need for large-scale, high-quality trials in this area. The review identifies effective techniques that could be deployed in future interventions.

## Introduction

Body image is the subjective “picture” that people have of their own body [1], regardless of how their body actually looks. Body image is a multifaceted construct, consisting of cognitive and affective components (i.e., how people think and feel about their body), perceptual components (i.e., how people perceive the size and shape of their body and body parts), and behavioural components (i.e., the actions that people perform for the purpose of checking on, tending to, altering, or concealing their body) [2]. *Negative body image* is expressed in one or more of the components of body image and is often characterised by a dissatisfaction with appearance and engaging in behaviours such as frequent self-weighing or mirror checking, or avoidance of public situations [3].

Studies have shown that negative body image can emerge in childhood. Approximately 50% of preadolescent girls and 30% of preadolescent boys dislike their body [4–6]. In adults, approximately 60% of women and 40% of men have a negative body image, and these rates remain stable across the lifespan [7,8]. Negative body image contributes to the development and maintenance of body dysmorphic disorder and eating disorders [9,10], and is associated with low self-esteem [11], depression [12], social anxiety [13], and impaired sexual functioning [14]. In addition, negative body image has serious consequences for health behaviours. For instance, negative body image predicts physical inactivity [15,16], unhealthy eating [9,17], and weight gain [18], and is associated with unsafe sex [19,20], smoking [21], and skin cancer risk behaviours [22].

### Interventions Designed to Improve Body Image

Given the associations between negative body image, psychological problems, and unhealthy behaviours, a large number of interventions have been designed to improve body image. The most prominent of these interventions is cognitive-behavioural therapy (CBT) [23,24]. Broadly speaking, CBT aims to help individuals to modify dysfunctional thoughts, feelings, and behaviours that contribute to negative body image. To achieve these improvements, a variety of cognitive and behavioural change techniques are used such as self-monitoring, cognitive restructuring, and exposure exercises.

Other interventions for improving body image can broadly be divided into four categories: fitness training, media literacy, self-esteem enhancement, and psychoeducation. Fitness training interventions include aerobic or anaerobic activities geared at improving physical capacities (e.g., muscular strength). Interestingly, objective improvements in fitness obtained by such interventions are inconsistently related to changes in body image. Instead, it appears that *perceived* improvements in physical capacities may play a more important role [25]. Fitness training interventions may also improve body image by encouraging individuals to focus more on the functionality of their body and less on their appearance, or by increasing their sense of self-efficacy [25,26].

The aim of media literacy interventions is to teach individuals to critically evaluate and challenge the images (e.g., of underweight women) and messages (e.g., that thin is beautiful) disseminated by the media that can cause negative body image [27–29]. In doing so, these images and messages are discredited and consequently their influence on body image should be reduced [29]. Examples of techniques used in media literacy interventions include educating individuals about the biased notion of beauty ideals that is perpetuated by the media and teaching strategies to reduce exposure to appearance-focused media.

Another set of interventions is designed to enhance self-esteem. The rationale for these interventions is that low self-esteem has been shown to predict negative body image, and thus, by improving how individuals feel about their overall worth, body image should improve as well [30]. Techniques used in such interventions focus on identifying and appreciating individual differences (e.g., in body shape, ethnicity), strengths (e.g., sense of humour, intelligence), and talents (e.g., singing, mathematics), and building skills that are necessary for healthy coping and development (e.g., interpersonal skills).

Finally, psychoeducation aims to teach individuals about issues related to negative body image including its causes and consequences [31]. Psychoeducation often includes information about the key features of a healthy lifestyle (e.g., physical activity), and is frequently combined with other types of interventions, such as self-esteem enhancement [32] or fitness training interventions [33]. It is important to note that there are additional approaches to improving body image that do not easily fit into these categories (CBT, fitness training, media literacy, self-esteem enhancement, or psychoeducation), such as evaluative conditioning [34–36] or mindfulness-based interventions [37]. However, these approaches are comparatively new and have not yet received as much empirical attention.

### How Effective are Interventions Targeted at Body Image?

Two narrative reviews have supported the efficacy of CBT [23,38], and Jarry and Ip’s [39] meta-analysis of 19 CBT interventions found a large, positive effect on body image (*d*_+_ = 1.00). In addition, Campbell and Hausenblas [40] found that fitness training interventions had a small effect on body image at posttest (*d*_+_ = 0.29), whereas Yager, Diedrichs, Ricciardelli, and Halliwell’s [41] review of classroom interventions (that used various intervention approaches) observed effect sizes in the small to medium range (*d*_*+*_ = 0.23 to 0.48). Based on these reviews, it seems that interventions designed to improve body image are effective, with effect sizes ranging from small (*d*_*+*_ = 0.23) to large (*d*_*+*_ = 1.00).

Three important issues concerning these reviews must be addressed, however. First, reviews to date have focused on the broad approach taken (e.g., CBT or fitness training) rather than the *specific change techniques* deployed in interventions. This may be problematic because interventions based on any single approach may use a variety of different change techniques related to that approach, and may also draw upon techniques from alternative approaches. For instance, CBT-based interventions may deploy any number of CBT-based techniques such as guided imagery or exposure exercises, discussion of the role of cognitions in body image, or teaching monitoring and restructuring of cognitions. One or more of these techniques could be responsible for the effectiveness of the CBT approach. Further, these CBT interventions might also involve techniques such as those related to media literacy or self-esteem enhancement. Analysing the specific change techniques or “active ingredients” [42] used in interventions targeted at body image is valuable because it helps to move research beyond the basic question of whether or to what extent interventions are effective, to address deeper questions about “*why* are interventions effective?” and “*what change techniques* best improve body image and warrant use in future interventions?”

Although the identification of change techniques in behavioural interventions is well established [42,43], to our knowledge, there is no taxonomy that can be used to characterise the techniques used in interventions targeted at body image. Therefore, as part of the present review, we developed a taxonomy of change techniques used in stand-alone interventions designed to improve body image. To generate the taxonomy, we drew upon both theoretical accounts of cognitive and behavioural change [44–46], Abraham and Michie’s [42] taxonomy of behavioural change techniques, and a careful analysis of the content of stand-alone interventions that targeted body image. The goal in developing the taxonomy was to combine top-down (theoretical) and bottom-up (empirical) approaches [47,48] in order to best characterise the specific change techniques used in intervention studies. The final taxonomy comprised 48 change techniques in six broad categories (see Table 1).

**Table 1:** Change Techniques Used in Stand-Alone Interventions to Improve Body Image.

Nr.	Label	Definition
*General cognitive-behavioural techniques for improving body image*		
1	Discuss cognitions and their role in body image	Discuss cognitions and the role that they play in feelings and behaviours that are related to body image. Attention should be paid to concepts such as irrational beliefs (e.g., that only beautiful people are successful), automatic thoughts, cognitive errors (e.g., dichotomous thinking), etc.
2	Teach self-monitoring and restructuring of cognitions	Teach participants techniques to monitor and restructure their cognitions. Monitoring and restructuring is often recorded in writing, for example, using a diary or log. Techniques that may be used include keeping thought records, using the A-B-C model (i.e., tracking the ‘activating event,’ one’s beliefs about the event, and the emotional or behavioural consequences of those beliefs), or the Triple Column Technique (i.e., recording one’s automatic thoughts, identifying the cognitive errors in those thoughts, and then responding critically and rationally to those thoughts).
3	Teach self-monitoring of behaviour	Teach participants to monitor and record their behaviour(s) as part of a behavioural change strategy. For example, participants may be asked to record the number of times they check their appearance in the mirror. Or, participants may be asked to record, using a diary, the number of pedometer-determined steps that they walk per day.
4	Change negative body language	Teach participants to improve the language they use to describe their body. For example, participants may be taught to avoid using negative, evaluative terms (e.g., “I have a disgusting belly”) and to instead use terminology that is nonjudgemental and fact-based (e.g., “I have a round belly”).
5	Shift focus on bodily attributes from negative to positive	Teach participants to focus their attention less on body parts they dislike and to focus more attention on other body parts and on seeing one’s body as a whole. This may also include teaching participants to focus less on appearance-related aspects of the body (e.g., weight, shape) and to focus more on functionality-related aspects of the body (e.g., bodily senses, movement).
6	Conduct guided imagery exercises	Focus and direct participants’ imagination, for example, by having participants relive an important event that influenced their body image or use their “mind’s eye” to look at parts of their body.
7	Conduct exposure exercises	Expose participants to their own body, or to a distressing body-image related situation, with the goal of gradually extinguishing negative reactions to these situations. For example, mirror exposure may be conducted to expose participants to their own body, or participants may be asked to exercise in public wearing form-fitting clothing.
8	Write about the body	Prompt participants to write about their body image. For example, participants may describe, in writing, their most distressing body parts or particular life events that influenced their body image.
9	Provide size-estimate exercises	Prompt participants to estimate the size of various body parts, for example using movable markers to indicate the width of their hips or by estimating the circumference of their waist. Provide participants with objective feedback on the accuracy of their estimates (e.g., by measuring the respective body part together with the participant) and have them repeat their estimates until they are accurate.
10	Prompt action-planning	Prompt detailed planning of the performance of a specific action (including context, frequency, duration, and intensity). The action may relate to behaviour (e.g., exercising), or cognition (e.g., engaging in positive self-talk). The context may be external (physical or social) or internal (physical, emotional, or cognitive experiences).
11	Teach time management skills	Teach participants skills to manage their time effectively, for example, by helping participants to schedule time to complete homework despite a busy schedule or to limit time spent engaging in undesired activities (e.g., watching too much television) and increase time spent engaging in desired activities (e.g., spending time with family).
12	Agree on a contract	Create and agree on a verbal or written contract specifying a specific response to be performed (and possibly, actions to overcome barriers) so that there is a record of participants’ resolution that is witnessed by another person (e.g., by a therapist or group member). The response may be behavioural (e.g., physical activity) or cognitive (e.g., engaging in positive self-talk).
13	Barrier identification	Identify barriers to performing a specific behaviour and plan ways of overcoming them. For example, participants may arrange a baby sitter so that they have alone-time to perform physical activity exercises. Or, participants may arrange weekly visits to a friend to counteract loneliness.
14	Provide performance feedback	Provide feedback about behaviour or performance on a task, for example, by giving participants feedback regarding their homework assignments or regarding the completion of mirror exposure.
15	Provide encouragement	Encourage participants regarding the (continued) performance of particular (cognitive or behavioural) responses, for instance, by encouraging participants to complete homework assignments or to continue progressing through the intervention.
16	Prompt identification as a role model	Indicate how participants may set a positive example for others and how they may positively influence others' thoughts, feelings, and behaviour. This technique may include indicating how participants can share the knowledge they learned in the intervention with others (e.g., by intervening when a friend engages in negative self-talk) and how they can use it to help others who are experiencing body image difficulties (e.g., offering advice to a friend that is afraid of social situations where her body is exposed, such as a pool party).
17	Teach relapse-prevention strategies	Teach strategies for when participants are confronted with perceived failures to cope with negative body image thoughts, feelings, or behaviours (e.g., purging after a meal). Identify the situations likely to result in participants readopting maladaptive cognitions and behaviours or failing to maintain adaptive cognitions and behaviours (e.g., meeting with appearance-focused friends), and help them plan to avoid or manage these situations (e.g., by practicing positive self-talk).
18	Provide stress-management training	Teach participants stress management techniques that do not target body image cognition and behaviour but that seek to reduce anxiety and stress. These techniques include progressive muscle relaxation, deep breathing, etc.
19	Identify alternative help resources	Notify participants of alternative help resources that they can access or utilise, such as self-help books, DVDs, or websites, or information about a psychologist or support group.
*Techniques for enhancing physical fitness*		
20	Provide physical activity exercises	Offer or lead physical activity exercises that participants can engage in (e.g., walking, aerobic dance, swimming, Pilates, etc.).
*Techniques providing media-literacy and promoting media resistance*		
21	Provide media literacy training	Provide media literacy training with the aim of helping participants to decipher media messages and to be critical of them. Key concepts may include: (1) media images are constructed by experts (e.g., clothing and lighting experts); (2) media images present only one version of reality; (3) the media influence how people feel about themselves; and (4) the purpose of media is to sell products, values, and ideas.
22	Discuss the beauty ideal	Discuss the concept of the beauty ideal, including topics such as the variation in the beauty ideal over time and across cultures, the unrealistic nature of the beauty ideal, the (false) assumptions made about the beauty ideal (e.g., if one is thin, one will be happy), etc.
23	Teach strategies for resisting the effect of the media	Teach participants strategies they can use to resist the impact of the media. For example, participants may be trained to focus on nonappearance aspects of models in advertisements (e.g., the activities they are engaged in), or they may decide to stop reading fashion magazines that feature extremely thin (female) or extremely muscular (male) models.
24	Provide media-critique exercises	Provide exercises that involve critiquing media images and the messages presented through them. For example, participants may be asked to generate arguments to counter the ‘thin is beautiful’ message presented in many advertisements, or they may be asked to examine stereotypes portrayed in music videos (e.g., that women are sexually passive).
25	Provide alternative images of women and/or men	Provide images of women and/or men (e.g., their faces, bodies) that are empowering and that go against the current beauty ideal. For example, provide participants with advertisements that promote positive body image (e.g., featuring people with a variety of body sizes and shapes) or show participants images that portray historical beauty ideals (e.g., Degas' painting *The Bather*).
*Techniques designed to enhance self-esteem*		
26	Discuss self-esteem	Discuss the concept of self-esteem, how self-esteem is formed, what factors influence self-esteem, how it relates to well-being, etc.
27	Provide self-esteem enhancement exercises	Provide exercises that aim to enhance the participants’ positive self-regard. For example, participants may write a list of their talents and positive personality traits or participants may practice giving each other compliments.
28	Discuss individual differences	Discuss the concept of individual differences regarding inner (e.g., personality) and outer (e.g., appearance) facets. Topics may include how individuals develop different traits, characteristics, and talents that make them unique, how individuals differ in appearance, body size, body shape, skin colour, etc.
29	Discuss alternatives to focusing on appearance	Discuss nonappearance-related aspects of the self and others. For example, discuss how the body can be viewed in terms of its functionality (e.g., fitness, sensory experience, health) or capacity to express internal qualities (e.g., kindness, intelligence, sense of humour) rather than in terms of appearance, or how mastery and pleasure can be achieved through the body (e.g., by receiving a massage or engaging in physical activities that one enjoys).
30	Discuss stereotypes	Discuss stereotypes, prejudice and discrimination related to gender or appearance. Topics may include stereotypes about women and men, stereotypes about thin or overweight people, the impact of prejudice and discrimination, etc.
31	Discuss age-related issues and challenges	Discuss age-related issues and challenges, as well as their impact on well-being. Topics may include the changes the body goes through during puberty, pregnancy, or menopause, different maturity rates, difficulties of navigating puberty and adolescence, etc.
32	Discuss interpersonal relations	Discuss interpersonal relations, for example, peer pressure, social rejection, the unacceptability and impact of appearance-based teasing, the effects of fat talk, how others may learn from one’s behaviour (e.g., social learning), etc.
33	Teach interpersonal skills	Teach participants interpersonal skills, such as how to communicate with others effectively, how to express one's opinion, how to resolve interpersonal conflicts, etc.
34	Discuss social comparisons	Discuss topics such as social comparison theory, the consequences of comparing one’s body with others’ bodies (e.g., friends, peers), the consequences of comparing one’s body with the beauty ideal, etc.
35	Provide social comparison exercises	Provide social comparison exercises with the primary aim to alter social comparison processes (either explicitly or implicitly). For example, participants may be asked to make nonappearance-based or downward social comparisons with models in fashion magazines (e.g., by writing down how one’s own body is more natural and authentic).
36	Provide a positive role-model	Provide participants with a role model, either real (e.g., another person who has experienced and conquered body image difficulties) or imaginary (e.g., a fictional character who demonstrates positive body image). Real role models can attend intervention sessions to talk to participants; real and imaginary role models can also be presented in written (e.g., in a story/description) form or in a film/video clip (e.g., an interview, movie).
*Techniques providing psychoeducation related to body image and healthy lifestyle*		
37	Discuss the concept of body image	Discuss the concept of body image, what body image is, and what are the different components of body image (e.g., evaluative, behavioural, perceptual).
38	Discuss the causes of negative body image	Discuss the causes and risk factors for negative body image (e.g., the beauty ideal, the tendency to make social comparisons, developmental events). These causes may be general (e.g., media influence) or specific (e.g., receiving a negative remark about one's weight), internal (e.g., perfectionism) or external (e.g., teasing).
39	Discuss the consequences of negative body image	Discuss the psychological consequences of negative body image, such as the development of an eating disorder, depression, low self-esteem, social anxiety, etc.
40	Discuss the behavioural expression of negative body image	Discuss how negative body image is expressed in various behaviours such as body checking (e.g., weighing, measuring, pinching, mirror checking), body avoidance (e.g., avoiding mirrors, wearing baggy clothing) or appearance preoccupation (e.g., time-consuming efforts to groom, manage, or alter appearance). This may also include discussing how these behaviours can be negative reinforcers (i.e., they may relieve distress in the short term, but maintain the problem in the long term).
41	Discuss healthy eating	Discuss healthy eating and nutrition, including topics such as guidelines for a balanced and healthy diet, how to read food labels and choose healthy foods, physiological cues (e.g., hunger, satiety), calories, fat, nutrients, vitamins, and the benefits of healthy eating for well-being.
42	Discuss physical activity	Discuss physical activity, such as various physical activities that can be engaged in, how to select physical activities that participants enjoy, and the benefits of physical activity for health and well-being.
43	Discuss eating pathology	Discuss eating disorders and related behaviours and cognitions, including topics such as risk factors for developing an eating disorder, unhealthy eating patterns (e.g., bingeing, fasting), dietary restraint, excessive exercising, and the consequences of eating pathology (e.g., fatigue).
44	Discuss stress	Discuss the concept of stress, what stress is (e.g., healthy vs. unhealthy forms), what causes stress, and what are the consequences of stress for health and well-being.
*Additional techniques for improving body image*		
45	Use evaluative conditioning	Use evaluative conditioning to alter implicit associations concerning the body. For example, in a computer task, pictures of the participants’ own body may be systematically paired with positive social feedback, or pictures of extremely thin models may be paired with words like "fake" and "unnatural."
46	Discuss feminism	Discuss topics regarding feminism, such as what it means to be feminist, misconceptions about feminism, feminist theories of body image and eating disturbance (e.g., the objectification theory), sex role conflicts, etc.
47	Discuss mindfulness	Discuss the concept of mindfulness, including aspects such as awareness, cognitive defusion, willingness to experience, accepting without judgment, and releasing the need for control. Discussions related to Acceptance and Commitment (e.g., pain as an unavoidable aspect of life) also fall under this category.
48	Provide mindfulness exercises	Provide mindfulness exercises, such as deep breathing, body scan, meditation, mindful eating, etc. Exercises related to Acceptance and Commitment (e.g., identification of values) or practicing gratitude also fall under this category.

Coders are encouraged to make note of any change techniques that do not fall into any of these categories.

Second, the present review also considers the issue of risk of bias both within individual studies and across studies. Risk of bias *within studies* refers to methodological features that could exaggerate the estimate of an intervention’s effectiveness [49]. The Cochrane Handbook of Systematic Reviews [50] has published a tool for assessing such bias, comprising seven domains such as incomplete outcome data (to assess attrition bias). Risk of bias *across studies* refers to factors that may affect the cumulative evidence obtained via meta-analysis. In particular, publication bias refers to the phenomenon that, compared to studies with nonsignificant results, those with significant results are more likely to be submitted and published (and, therefore, are more likely to be included in systematic reviews, [51]). The strategy for assessing publication bias recommended by the Cochrane Handbook of Systematic Reviews [50] is to generate a funnel plot and test for asymmetry using Egger’s regression [52]; if the regression coefficient is significant, the *trim and fill* procedure [53] can be used to correct for asymmetry in the funnel plot arising from publication bias.

Related to publication bias is the phenomenon of *small sample bias*: the tendency for estimates of the intervention effect to be more favourable in smaller studies. Coyne, Thombs, and Hagedoorn [54] recently critiqued interventions in the field of behavioural medicine for over-relying on small, underpowered trials [55,56]. Coyne et al. [54] recommended that meta-analysts correct for small sample bias by estimating intervention effects separately for studies that contain at least 35 participants per cell, and thus have ≥ 55% power to detect an effect of medium magnitude. Only Campbell and Hausenblas [40] reported a funnel plot, Egger’s regression, and trim and fill analysis (as well as the Fail Safe *N*, [51]), and none of the previous meta-analyses have tested or corrected for small sample bias or assessed risk of bias within individual studies. Consequently, the results of prior reviews could exhibit biases that overestimate the effect of interventions on body image [49].

Third, although previous reviews excluded studies without a control condition, many of the included studies did not randomly assign participants to conditions [23,40,41] or did not include a pretest measure of body image [23]. According to the Cochrane Handbook of Systematic Reviews [50], randomisation is “the only way to prevent systematic differences between baseline characteristics of participants in different intervention groups in terms of both known and unknown (or unmeasured) confounders” ([57], p. 90). Pretest-posttest designs are important because they increase the power and precision of statistical tests (as each participant serves as his or her own control) and offer the best estimate of *improvement* (i.e., positive change) due to the intervention [58–60].

### The Present Meta-Analysis

The aims of this meta-analysis were to (a) quantify the effectiveness of stand-alone interventions on body image taking account of the risk of bias both within and across studies, and (b) identify the specific change techniques that are associated with improvements in body image. There were four inclusion criteria for the review. First, the intervention to improve body image had to be stand-alone. We followed Jarry and colleagues’ [38,39] precedent in reviewing stand-alone body image interventions and used their definition of treatment: “A stand-alone body image treatment was defined as one where the body image intervention was not combined with another extensive psychological therapy. Therefore, studies where body image therapy was part of a comprehensive eating disorder treatment were excluded” ([38], p. 320). Jarry and Ip [39] pointed out that “Interventions for BI [body image] disturbance are often imbedded in larger eating disorder treatment programs [61], which complicates the assessment of their effectiveness” (p. 317). Thus, to meet this criterion, interventions had to have body image improvement as their primary and ultimate goal. This focus on stand-alone interventions should serve to reduce heterogeneity of effect sizes and enhance the interpretability of findings [57].

The second criterion was that studies had to include a control group. Third, participants had to be randomly assigned to either the intervention or control group. Finally, at least one pretest and one posttest measure of body image had to be taken. Body image was the primary outcome variable, but the effects of interventions on two secondary variables related to vulnerability for developing negative body image–internalisation of the beauty ideal and the tendency to make social comparisons–were also included as outcomes [62,63]. Features related to the sample, intervention, and methodology were assessed as potential moderators of intervention effects.

## Method

### Literature Search and Study Selection

Five strategies were used to generate the sample of studies: (a) we conducted computerised searches of the databases PsychINFO (1935 –Present), PubMed (1952 –Present), and Web of Science (1988 –Present) using the terms *body anxiety* or *body attitudes* or *body checking* or *body concern* or *body esteem* or *body evaluation* or *body dissatisfaction* or *body image* or *body image disturbance* or *body satisfaction* or *body shame* or *body surveillance* AND *campaign* or *experiment* or *initiative* or *intervention* or *prevention* or *technique* or *treatment* or *trial* or *strategy*; (b) we reviewed the reference lists of previous reviews; (c) we looked at the reference lists of all included papers (i.e., an ancestry approach) [64]; (d) we sent requests for relevant studies to the mailing lists of nine major societies (Association for Behavioral and Cognitive Therapies, European Association for Behavioural and Cognitive Therapies, European Association of Social Psychology, Eating Disorders Research Society, European Health Psychology Society, Obesity Society, Society of Experimental Social Psychology, Social Personality and Health Network, and Society for Personality and Social Psychology); and (e) we e-mailed established researchers working in the field to request studies. In particular, we e-mailed Thomas Cash, Rachel Calogero, Alex Clarke, Catherine Cook-Cottone, Alexandra Corning, Janis Crowther, Sigrun Danielsdottir, Nova Deighton-Smith, Helga Dittmar, Barbara Fredrickson, Ann Frisén, Shelly Grabe, Sarah Grogan, Heather Hausenblas, Kristina Holmqvist-Gattario, Michael Levine, Kristine Luce, Traci Mann, Kathleen Martin Ginis, Marita McCabe, Taryn Myers, Dianne Neumark-Sztainer, Jennifer O’Dea, Susan Paxton, Adria Pearson, Thomas Pruzinsky, Lina Ricciardelli, Danielle Ridolfi, Giuseppe Riva, James Rosen, Marlene Schwartz, Roz Shafran, Linda Smolak, Eric Stice, Viren Swami, Kevin Thompson, Marika Tiggemann, Tracy Tylka, David Veale, Tracey Wade, Zali Yager, and Patricia van den Berg. In addition, Michael Levine forwarded our request for unpublished research to his personal mailing list of approximately 115 researchers who are actively involved in body image research.

The last search was conducted on March 2^nd^, 2015. No date or publication status restrictions were imposed, but only English-language studies were eligible (to allow independent assessment of the details of all interventions and change techniques included in the meta-analysis). The first author screened the records (i.e., title and abstract) obtained from the literature search twice; if the record indicated that the research involved an intervention and body image was measured, then the full-text article was consulted. If the full-text article did not provide sufficient information to determine eligibility (according to the inclusion criteria) or to calculate effect sizes, then all authors of the respective studies were e-mailed (authors’ up-to-date contact information was obtained via online searches). If the authors did not respond after three attempts, then the study was excluded.

### Effect Size Estimation

The primary outcome was body image and the secondary outcomes were beauty ideal internalisation and the tendency to make social comparisons. We calculated Cohen’s effect size *d* for each outcome using Morris’ [60] recommended method for computing effect sizes in pretest-posttest control group designs: The mean pre-posttest change of the control group was subtracted from the mean pre-posttest change of the experimental group, and was then divided by the pooled pretest standard deviation; a bias adjustment for sample size was also applied [60]. The first author and a research assistant independently calculated the effect sizes and sample sizes using separate data extraction sheets. The mean difference between the two sets of effect sizes was 0.001; sample size calculations were identical.

The following factors were taken into account when calculating the effect sizes. Where measures of an outcome were taken at two or more time points following the intervention, we used the longest-term follow-up measurement to calculate the effect sizes to permit a strict test of intervention effects [65]. When both intention-to-treat and completer-only analyses were conducted, we calculated effect sizes using the intention-to-treat data to reduce the impact of attrition bias. When multiple measures of an outcome were available, we computed the average effect size within each study to ensure independence. For the same reason, we divided the sample size for the control group by the number of intervention groups when studies included more than one intervention [66]. When studies employed a crossover design, participants who first received the intervention were considered the intervention group, whereas participants who first received the control intervention were considered the control group, and we excluded the data from the second phase of such studies (i.e., when participants switched conditions). Effect sizes were interpreted using Cohen’s [67] guidelines where *d*_+_ = 0.20, 0.50, and 0.80 constitute small, medium, and large effects, respectively.

### Recorded Variables

#### Change techniques

Descriptions of the interventions provided in the original reports were analysed, and generated a taxonomy that comprised 48 change techniques (see Table 1). Techniques could be classified in six broad categories: (a) general cognitive-behavioural techniques for improving body image (e.g., discuss cognitions and their role in body image); (b) techniques for enhancing physical fitness (e.g., provide physical activity exercises); (c) techniques providing media literacy and promoting media resistance (e.g., provide media critique exercises); (d) techniques designed to enhance self-esteem (e.g., discuss individual differences); (e) techniques providing psychoeducation related to body image and healthy lifestyle (e.g., discuss the causes of negative body image); and (f) additional techniques for improving body image (e.g., use evaluative conditioning). For all intervention conditions, the presence versus absence of each technique was coded (0 = *absent*, 1 = *present*) so that the association between deployment of particular change techniques and effects on body image could be assessed via meta-regression.

#### Risk of bias within individual studies

Risk of bias within individual studies was assessed using the Cochrane Collaboration’s Tool for Assessing Risk of Bias [50], which involves rating each study in seven domains: random sequence generation, allocation concealment, blinding of participants and personnel, blinding of outcome assessment, incomplete outcome data, selective reporting of outcomes, and “other sources of bias” (i.e., any remaining concerns about potential sources of bias that are not covered by the prior categories). Each intervention was coded as high, low, or unclear risk of bias with regard to each of the seven domains. A code of unclear risk of bias is used when insufficient information is provided to confer a judgement of either high or low risk. A summary assessment was also made for each intervention based on Higgins and Green’s [50] guidelines. It is important to note that we coded blinding of participants, not personnel, because it would be impossible for all personnel to be blinded to the participants’ condition (e.g., when administering an intervention). Blinding of outcome assessment also concerned participants because the present outcomes are all self-reported outcomes [68].

#### Moderator variables

The moderator variables related to characteristics of the sample, intervention, and methodology. Studies that screened participants for having a negative body image were considered selected. Studies that delivered interventions in classroom settings or where participants were not screened for having a negative body image were considered nonselected. Interventions were divided into those that targeted participants at childhood (12 years and younger), adolescence (13 to 17 years), as well as early (18 to 29 years), middle (30 to 64 years), and late (65 years and older) adulthood [4,8,69,70]. Gender was coded as the percentage of female participants in the sample.

Intervention format was coded as individual (self-administered or delivered to one person) or group. We coded the presence versus absence of a facilitator, and whether the intervention comprised a single session or multiple sessions. The nature of the control group was coded as either active (i.e., where participants received a placebo intervention) or passive (i.e., where participants received no intervention or were placed on a waiting list). Time to follow-up was categorised into three levels [39]: posttest only, short-term follow-up (3 months or less), or longer-term follow-up (longer than 3 months).

#### Reliability of codings

The first and fourth author independently coded each intervention. Reliability was assessed using kappa adjusted for prevalence and bias [71] because values were generally unbalanced across the two code options (i.e., technique present vs. absent). Kappas ranged from 0.68 to 1.00 (*Mdn* = .90); discrepancies were resolved by discussion.

### Meta-Analytic Strategy

All of the analyses were pre-specified and conducted using STATA (Release 11). Although we followed a pre-specified plan for conducting the present meta-analysis, the protocol was not registered as we were not aware that this was feasible when the review started. We used a random effects model to calculate the sample-weighted average effect sizes because studies were likely to be “different from one another in ways too complex to capture by a few simple study characteristics” ([72], p. 526), and because random effects models enhance the generalisability of meta-analytic findings [73].

The impact of risk of bias within individual studies was tested by estimating the effect sizes for interventions deemed high risk, low risk, and unclear risk, and by comparing these effect sizes using the *Q* statistic. Publication bias was assessed using several procedures, as recommended by Field and Gillet [73]. First, the data were Winsorised using both the 90^th^ and the 80^th^ percentiles to determine how studies with the smallest and largest effect sizes influenced the overall effect size. Second, to facilitate comparability with prior reviews, we calculated the Fail Safe *N* (FSN, [51]), which is the number of additional ‘negative’ studies (studies in which the intervention effect was zero) that would be needed to increase the *p*-value for the sample-weighted average effect to above 0.05. We used Rosenthal’s [51] recommended tolerance level of 5*k* + 10 (where *k* is the number of independent tests): If the FSN exceeds the tolerance level, the findings are considered resistant to publication bias. Third, we compared the effect sizes for published vs. unpublished studies to assess the impact of publication status. Fourth, we created a funnel plot (a scatterplot of each effect size against its standard error, [74]); visual inspection of the plot indicates where studies are ‘missing’ (usually studies with negative or null effects). To formally test funnel plot asymmetry, we used Egger’s regression [52], which regresses the intervention effect estimate on its standard error, weighted by the inverse of the variance of the intervention effect estimate.

Fifth, if Egger’s regression proved significant, the *trim and fill* procedure [53] was used. The basis of the procedure is to (1) ‘trim’ (remove) the smaller studies causing funnel plot asymmetry, (2) use the trimmed funnel plot to estimate the true ‘centre’ of the funnel, then (3) replace the omitted studies and their missing ‘counterparts’ around the centre (‘filling’). As well as providing an estimate of the number of missing studies, the trim and fill procedure provides an adjusted intervention effect by performing a meta-analysis including the filled studies. We corrected for small sample bias using the procedure recommended by Coyne et al. [54]: We computed the average effect size in studies with at least 35 participants per condition.

Variability in the effect sizes for body image and the secondary outcomes was calculated using the *Q* and *I* ^2^ statistics. We used meta-regression to test the association between change techniques and effect sizes whenever *k* ≥ 4 –the criterion proposed by Michie, Abraham, Whittington, McAteer, and Gupta [75]. Meta-regression was also used to test the association between gender and the effect of the interventions on body image. The other potential moderators of intervention effects involved mutually exclusive categories. We therefore estimated an effect size for each level of the moderator whenever *k* ≥ 4, using the *Q* statistic to test the difference between the effect sizes.

## Results

### Study Selection and Characteristics

**Fig 1:**
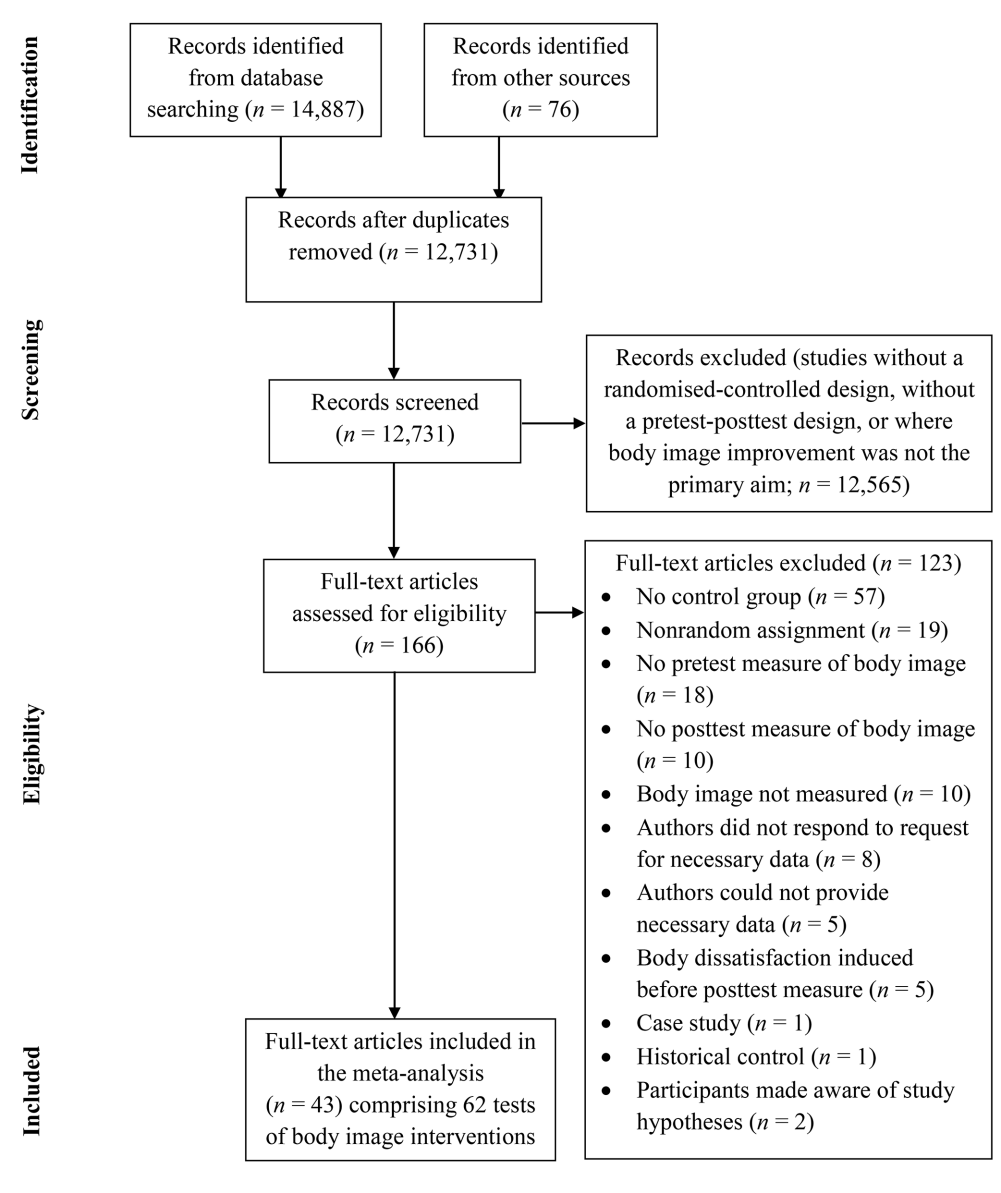
Flow of Studies through the Current Meta-Analysis.

Fig 1 presents the flow of studies through the review. The literature search returned 12,731 English language records (after duplicates were removed). In total, 166 full-text articles were assessed for eligibility. Forty-three studies were included in the meta-analysis, providing 62 tests of stand-alone interventions to improve body image, with a total sample size of *N* = 3,846. The studies were published between 1987 and 2015, and were conducted in the United States (*n* = 28), Australia (*n* = 10), the Netherlands (*n* = 8), Turkey (*n* = 8), the United Kingdom (*n* = 5), Canada (*n* = 1), Portugal (*n* = 1), and Sweden (*n* = 1). Table 2 presents the 62 interventions, their effect sizes, and the measures used to calculate respective effect sizes.

**Table 2:** Effect Sizes for Studies Included in the Meta-Analysis.

			Effect size categories					
			Body image		Beauty ideal internalisation		Social comparison tendencies	
Authors	*N*c	*N*e	*d*_+_ (*95% CI)*	Measures	*d*_+_ (*95% CI)*	Measures	*d*_+_ (*95% CI)*	Measures
Albertson et al. (2014)	130	98	0.37 (0.10 to 0.63)	3, 18				
Alleva et al. (2014)–Study 1^a^	20	18	0.08 (-0.56 to 0.71)	12				
Alleva et al. (2014)–Study 1^b^	22	19	0.58 (-0.05 to 1.21)	12				
Alleva et al. (2014)–Study 2	39	41	-0.05 (-0.49 to 0.39)	12				
Alleva et al. (2015)	40	41	0.33 (-0.11 to 0.77)	3, 26, 27				
Arbour & Ginis (2008)	17	25	0.64 (0.01 to 1.27)	1				
Asci (2002)^a^	36	37	0.33 (-0.14 to 0.79)	32				
Asci (2002)^b^	32	33	0.41 (-0.09 to 0.90)	32				
Asci (2003)	20	20	0.22 (-0.40 to 0.84)	31				
Asci et al. (1998)^c^	7.5^y^	15	0.46 (-0.42 to 1.35)	21, 32				
Asci et al. (1998)^d^	7.5^y^	15	0.37 (-0.51 to 1.26)	21, 32				
Bhatnagar (2013)	19	19	0.78 (0.12 to 1.44)	10, 22, 26, 27				
Burgess et al. (2006)	25	25	2.06 (1.38 to 2.75)	2, 23				
Butters & Cash (1987)	16	15	1.42 (0.63 to 2.20)	15, 16, 20			-1.38 (-2.16 to -0.59)	2
Corning et al. (2010)	16	15	0.51 (-0.20 to 1.23)	13, 14, 24				
Cousineau et al. (2010)	98	92	-0.19 (-0.48 to 0.10)	7, 8, 40				
Cruz-Ferreira et al. (2011)	24	38	0.19 (-0.32 to 0.70)	33, 34				
Delinsky & Wilson (2006)	20	21	0.25 (-0.36 to 0.87)	4, 10, 35				
Divsalar (2006)^e^	11^y^	22	0.21 (-0.52 to 0.94)	22, 26, 27	-0.32 (-1.05 to 0.41)	2		
Divsalar (2006)^f^	11^y^	22	0.002 (-0.72 to 0.73)	22, 26, 27	-0.12 (-0.84 to 0.61)	2		
Dohnt & Tiggemann (2008)	42	42	-0.33 (-0.76 to 0.10)	42	-0.25 (-0.68 to 0.18)	1		
Duncan et al. (2009)^b^	17	17	0.09 (-0.59 to 0.76)	9				
Duncan et al. (2009)^a^	18	16	0.48 (-0.20 to 1.16)	9				
Dunigan et al. (2011)	26	23	0.36 (-0.20 to 0.93)	12				
Earnhardt et al. (2002)	25	23	-0.13 (-0.70 to 0.44)	5				
Emerson (1995)	20	20	0.33 (-0.30 to 0.95)	6				
Fisher & Thompson (1994)^g^	8^y^	16	0.46 (-0.40 to 1.32)	10, 24, 29, 30				
Fisher & Thompson (1994)^h^	8^y^	14	0.70 (-0.19 to 1.60)	10, 24, 29, 30				
Gehrman et al. (2006)^a^	19	33	0 (-0.56 to 0.56)	24				
Gehrman et al. (2006)^b^	16	16	0.07 (-0.63 to 0.76)	24				
Geraghty et al. (2010)^i^	115.5^y^	130	0.24 (-0.01 to 0.49)	26, 27				
Geraghty et al. (2010)^j^	115.5^y^	118	0.14 (-0.11 to 0.40)	26, 27				
Grasso (2007)	98	83	-0.06 (-0.36 to 0.23)	11, 26, 38				
Heinicke et al. (2007)	37	36	0.62 (0.15 to 1.09)	19	-0.38 (-0.84 to 0.09)	2	-0.47 (-0.94 to -0.01)	1
Jansen et al. (2008)	8	8	0.69 (-0.32 to 1.70)	43				
Lew et al. (2007)	45	50	0.27 (-0.13 to 0.68)	25, 28, 29, 37				
Lindwall & Lindgren (2005)	35	27	0.18 (-0.19 to 0.56)	32, 39				
Martijn et al. (2012)—Study 2	19	17	0.40 (-0.26 to 1.07)	12				
Martijn et al. (2010)^k^	14	14	0.46 (-0.29 to 1.21)	41, 42				
Martijn et al. (2010)^l^	14	12	0.07 (-0.71 to 0.84)	41, 42				
McCabe et al. (2006)^a^ ^,^ ^m^	33	41	-0.37 (-0.84 to 0.09)	44, 45				
McCabe et al. (2006)^a^ ^,^ ^n^	48	51	-0.09 (-0.48 to 0.31)	44, 45				
McCabe et al. (2006)^b^ ^,^ ^m^	36	44	0.20 (-0.25 to 0.64)	44, 45				
McCabe et al. (2006)^b^ ^,^ ^n^	51	64	0.01 (-0.36 to 0.38)	44, 45				
McLean et al. (2011)	29	32	1.51 (0.94 to 2.08)	10, 18	-1.07 (-1.61 to -0.53)	3	-0.90 (-1.43 to -0.38)	3
Murphy (1994)^k^	6	7	0.62 (-0.50 to 1.74)	10, 18, 24				
Murphy (1994)^l^	7	8	0.36 (-0.66 to 1.39)	10, 18, 24				
Özdemir et al. (2010)^o^	4^y^	11	0.92 (-0.28 to 2.11)	32				
Özdemir et al. (2010)^p^	4^y^	12	0.46 (-0.68 to 1.60)	32				
Özdemir et al. (2010)^q^	4^y^	11	0.88 (-0.31 to 2.07)	32				
Paxton et al. (2007)^r^	18.5^y^	42	0.95 (0.38 to 1.52)	10, 18	-0.55 (-1.11 to 0.002)	3	-0.74 (-1.31 to -0.18)	3
Paxton et al. (2007)^s^	18.5^y^	37	0.40 (-0.16 to 0.97)	10, 18	-0.29 (-0.86 to 0.27)	3	-0.41 (-0.98 to 0.15)	3
Pearson et al. (2012)	39	34	0.57 (0.10 to 1.04)	28, 29				
Peterson et al. (2006)^t^	23.5^y^	51	0.30 (-0.19 to 0.80)	42				
Peterson et al. (2006)^u^	23.5^y^	49	0.03 (-0.46 to 0.53)	42				
Ridolfi & Vander Wal (2008)	39	42	0.21 (-0.22 to 0.65)	19	-0.03 (-0.47 to 0.40)	3		
Rosen et al. (1995)^v^	23	25	1.67 (1.02 to 2.33)	18				
Rosen et al. (1995)^w^	27	27	2.38 (1.69 to 3.08)	18				
Rosen et al. (1989)	10	13	1.40 (0.49 to 2.32)	18, 24, 36				
Stanford & McCabe (2005)	69	52	0.22 (-0.14 to 0.58)	17				
Waggoner (1999)^g^	3.5^y^	8	0.55 (-0.73 to 1.83)	10, 24				
Waggoner (1999)^x^	3.5^y^	8	0.46 (-0.81 to 1.73)	10, 24				

*N*_c_ = Number of participants in the control condition; *N*_*e*_ = Number of participants in the experimental condition; *d*_+_ = sample-weighted average effect size; *95% CI* = 95% confidence interval.

^a^ Females.

^b^ Males.

^c^ Dance aerobics.

^d^ Step aerobics.

^e^ Video Intervention 1.

^f^ Video Intervention 2.

^g^ Cognitive-behavioural therapy (CBT).

^h^ Fitness training intervention.

^i^ Gratitude diaries.

^j^ Monitoring and restructuring.

^k^ High-risk women.

^l^ Low-risk women.

^m^ 3^rd^ and 4^th^ grade students.

^n^ 5^th^ and 6^th^ grade students.

^o^ Cycling.

^p^ Running.

^q^ Swimming.

^r^ Face-to-face intervention.

^s^ Internet intervention.

^t^ Feminist intervention.

^u^ Psychoeducation intervention.

^v^ Rosen, Orosan, & Reiter [76].

^w^ Rosen, Reiter, & Orosan [77].

^x^ Cognitive therapy.

^y^ To accommodate testing for two experimental conditions, the sample size of the control group has been divided by two.

Measures of body image are coded as follows: 1 = Adult Body Satisfaction Questionnaire (ABSQ, [78]): Satisfaction with Physical Appearance Subscale; 2 = Body Attitudes Questionnaire (BAQ, [79]); 3 = Body Appreciation Scale (BAS, [80]); 4 = Body Checking Questionnaire (BCQ, [81]); 5 = Body Esteem Scale (BES, [82]); 6 = BES [82]: Sexual Attractiveness Subscale; 7 = Body Esteem Scale for Adolescents and Adults (BES, [83]): Appearance Body Esteem Subscale; 8 = BES [83]: Weight Body Esteem Subscale; 9 = Body Esteem Scale for Children [84]; 10 = Body Image Avoidance Questionnaire (BIAQ, [85]); 11 = Body Image Disturbance Questionnaire (BIDQ, [86]); 12 = Body Image States Scale (BISS, [87]); 13 = Body Parts Dissatisfaction Scale (BPDS, [88]): Number of Body Parts Wished Smaller; 14 = BPDS [88]: Number of Body Parts with Which Content; 15 = Body Parts Satisfaction Scale (BPSS, [89]): Body Parts Satisfaction Subscale; 16 = BPSS [89]: Overall Appearance Satisfaction Subscale; 17 = Body Satisfaction and Body Change Inventory (BSBCI, [90]): Body Satisfaction Subscale; 18 = Body Shape Questionnaire (BSQ, [91]); 19 = Body Shape Questionnaire- Short Form (BSQ-SF, [92]); 20 = Body-Self Relations Questionnaire (BSRQ, [93]): Appearance Evaluation Subscale; 21 = Berscheid, Walster, and Bohrnstedt Body Image Questionnaire [89]; 22 = Contour Drawing Rating Scale (CDRS, [94]); 23 = Child and Youth Physical Self-Perception Profile (CY-PSPP, [95]): Body Attractiveness Subscale; 24 = Eating Disorders Inventory–II (EDI-II, [96]): Body Dissatisfaction Subscale; 25 = Figure Rating Scale (FRS, [97]); 26 = Multidimensional Body-Self Relations Questionnaire (MBSRQ, [98]): Appearance Evaluation Subscale; 27 = MBSRQ [98]: Body Areas Satisfaction Subscale; 28 = Physical Appearance State and Trait Anxiety Scale (PASTAS, [99]: State Nonweight Subscale; 29 = PASTAS [99]: State Weight Subscale; 30 = PASTAS [99]: Trait Weight Subscale; 31 = Marsh Physical Self-Description Questionnaire (PSDQ, [100]); 32 = Physical Self-Perceptions-Inventory (PSPP, [101]): Bodily Attractiveness Subscale; 33 = Physical Self-Concept Scale (PSS, [102]): Perception of Appreciation of Physical Appearance Subscale; 34 = PSS [102]: Perception of Physical Appearance Subscale; 35 = Satisfaction with Body Parts Scale (SBPS, [89]); 36 = Self-Report Behavioral Avoidance Questionnaire [103]; 37 = Self-reported current weight = self-reported ideal weight; 38 = Situational Inventory of Body Image Dysphoria–Short Form (SIBID-S, [104]); 39 = Social Physique Anxiety Scale (SPAS, [105]); 40 = Self-Perception Profile for Adolescents [106]: Physical Appearance Subscale; 41 = State Self-Esteem Scale (SSES, [107]): Appearance Subscale; 42 = Visual Analogue Scales (VAS) to assess body satisfaction; 43 = VAS to assess feelings of beauty; 44 = VAS to assess muscle dissatisfaction [108]; 45 = VAS to assess weight dissatisfaction [108].

Measures of beauty ideal internalisation are coded as follows: 1 = Questions about desire to look like TV and pop stars; 2 = Sociocultural Attitudes Toward Appearance Questionnaire (SATAQ, [109]): Internalization of the Thin Ideal Subscale; 3 = Sociocultural Attitudes Toward Appearance Questionnaire-III (SATAQ-III, [110]): General Internalization Subscale.

Measures of social comparison tendencies are coded as follows: 1 = Body Comparisons Scale (BCS, [111]); 2 = Physical Appearance Beliefs Test (PABT, [112]): Social Comparisons Subscale; 3 = Physical Appearance Comparison Scale (PACS, [113]).

### Overall Intervention Effect Sizes

Table 3 shows the overall effect of the interventions on the primary and secondary outcomes. The sample-weighted improvement in body image was of small-to-medium magnitude (*d*_+_ = 0.38) and was reliable (i.e., the confidence interval did not contain zero). The sample-weighted effect sizes for internalisation of the beauty ideal (*d*_+_ = -0.37) and the tendency to make social comparisons (*d*_+_ = -0.72) were of small-to-medium and large magnitude, respectively, and were both reliable. Thus, the interventions appear to be effective in improving body image and reducing internalisation of the beauty ideal and the tendency to make social comparisons.

**Table 3:** Overall Effect of Interventions on Outcomes.

Outcome	*N*	*k*	*d*_+_ (*95% CI)*	*Q*	*I* ^2^
Body image	3,846	62	0.38 (0.27 to 0.50)	176.26***	65.4
Beauty ideal internalisation	481	8	-0.37 (-0.60 to -0.15)	10.12	30.8
Social comparison tendencies	281	5	-0.72 (-1.01 to -0.43)	5.38	25.7

*k* = number of effect sizes; *d*_*+*_ = sample-weighted average effect size; *95% CI* = 95% confidence interval; *Q* = homogeneity *Q* statistic; *I* ^*2*^ = homogeneity *I* ^*2*^ statistic.

*** *p* < 0.001.

### Risk of Bias within Individual Studies

S2 Table shows the risk of bias for each intervention. The majority of studies did not specify how participants were randomly allocated to condition (*k* = 43), and whether this allocation was adequately concealed (*k* = 47). Studies were similarly divided according to those where participants were not blinded (*k* = 30) vs. blinded (*k* = 29) to the knowledge of their allocated condition, and in the majority of studies outcome assessment was not blinded (*k* = 35). Risk of attrition bias was low in most studies (*k* = 42), as were “other sources of bias” (*k* = 48). The other sources of bias concerned differences between groups at baseline (e.g., in body dissatisfaction) that were either statistically significant (high risk; *k* = 2) or not statistically checked (unclear risk; *k* = 12). All of the interventions were coded as having unclear risk of bias with regard to selective reporting of outcomes–a finding that is common in systematic reviews [50]. To facilitate comparisons between studies, we therefore did not incorporate this domain when calculating the summary assessment. The summary assessments indicated that 40 studies exhibited high risk of bias whereas the remaining 22 studies had unclear risk of bias. Studies that had high risk of bias produced significantly larger improvements in body image (*d*_+_ = 0.44; *95% CI* = 0.29 to 0.59) compared to studies that had unclear risk of bias (*d*_+_ = 0.29; *95% CI* = 0.10 to 0.48), *Q*(1) = 4.29, *p* = 0.03. Only one study that assessed internalisation of the beauty ideal, and no studies that assessed social comparison tendencies, had unclear risk of bias, so comparisons could not be conducted for these outcomes.

### Risk of Bias across Studies

Next, we undertook tests of, and corrections for, publication bias and small sample bias (Table 4). Using 90^th^ and 80^th^ percentile Winsorisation, the effects of the interventions on body image were, respectively, *d*_+_ = 0.37 (*95% CI* = 0.26 to 0.47) and *d*_+_ = 0.34 (*95% CI* = 0.25 to 0.43). These values are similar to the overall effect size (*d*_+_ = 0.38), suggesting that the largest and smallest effects did not bias the results. The FSN indicated that 2,282 unpublished studies with zero effect sizes would need to exist in order to invalidate the finding that the interventions improved body image. This value exceeds the tolerance value of 320 studies and suggests that the findings are resistant to publication bias.

**Table 4:** Tests for Publication Bias and Small Sample Bias.

		Outcome		
Procedure		Body image	Beauty ideal internalisation	Social comparison tendencies
*Winsorisation*				
80^th^ percentile				
	*d*_*+*_ (*95% CI*)	0.34 (0.25 to 0.43)	NA	NA
90^th^ percentile				
	*d*_*+*_ (*95% CI*)	0.37 (0.26 to 0.47)	NA	NA
*Fail Safe N*				
	Fail Safe N (tolerance value)	2,282 (320)	36 (50)	56 (35)
*Publication status*				
Published				
	*k*	52	6	5
	*d*_*+*_ (*95% CI*)	0.40 (0.27 to 0.54)	-0.41 (-0.69 to -0.13)	-0.72 (-1.01 to -0.43)
Unpublished				
	*k*	10	2	NA
	*d*_*+*_ (*95% CI*)	0.19 (0.004 to 0.38)	-0.22 (-0.73 to 0.29)	NA
*Q*		4.45*	0.44	NA
*Egger’s regression*				
	β (*SE*)	1.91 (0.51)***	-1.06 (2.47)	-5.02 (2.45)
*Trim and fill analyses*				
	Imputed (*k*)	21	NA	NA
	*d*_*+*_ (*95% CI*)	0.15 (0.02 to 0.28)	NA	NA
*Adequately powered studies*				
	*k*	16	3	1
	*d*_*+*_ (*95% CI*)	0.13 (0.02 to 0.24)	-0.21 (-0.47 to 0.04)	-0.47 (-0.94 to -0.01)

*d*_*+*_ = sample-weighted average effect size; *95% CI* = 95% confidence interval; *k* = number of effect sizes; *Q* = homogeneity *Q* statistic; β = beta from Egger’s regression; *SE* = standard error; NA = not applicable (because Egger’s regression was not significant or because there were too few tests to permit computation of average effect size).

* *p* < 0.05

*** *p* < 0.001.

**Fig 2:**
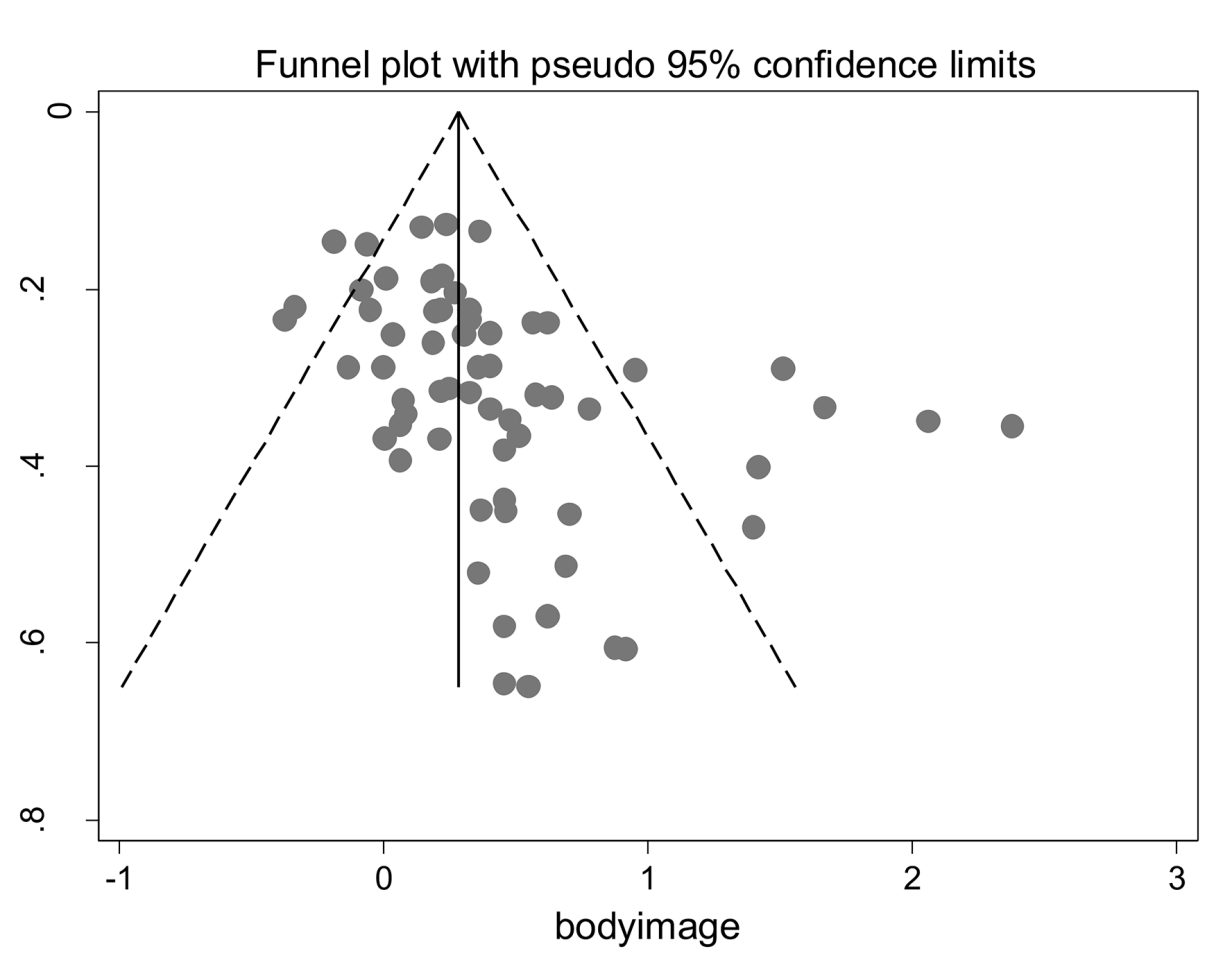
Funnel Plot of Effect Sizes (d_+_) for Body Image. s.e. = standard error.

However, more stringent tests of publication bias [50] offered a different conclusion. Sixteen percent of the studies included in the review (*k* = 10) were unpublished. The effect size from these studies (*d*_+_ = 0.19, *95% CI* = 0.004 to 0.38) was significantly smaller than the effect size derived from published studies (*d*_+_ = 0.40, *95% CI* = 0.27 to 0.54. *k* = 52), *Q*(1) = 4.45, *p* = 0.035 (though the percentage of unpublished [70%] vs. published [63.5%] studies at high risk of bias was similar). Furthermore, the funnel plot for body image effect sizes was asymmetrical, with studies reporting negative or zero effect sizes being absent (Fig 2). Egger’s regression was significant (*p* < 0.001) and indicative of publication bias in the distribution of effect sizes. Trim and fill analysis imputed 21 additional effect sizes, resulting in an overall effect size of *d*_+_ = 0.15 (*95% CI* = 0.02 to 0.28). Only 16 out of the 62 studies (26%) had at least 55% power to detect a medium effect. Correction for small sample bias showed that the effect size for interventions with at least 35 participants per condition was *d*_+_ = 0.13 (*95% CI* = 0.02 to 0.24). In sum, the overall effect size estimate of *d*_+_ = 0.38 for improved body image appears to be inflated by publication bias and small sample bias. Findings from unpublished studies, adequately powered studies, and trim and fill analyses all converge on the conclusion that the overall effect of interventions on body image is of small magnitude (*d*_+_ = 0.13 to 0.19), yet still reliable.

With regard to the secondary outcomes, FSN suggested the presence of publication bias in tests of internalisation of the beauty ideal, and effects were not reliable in the two unpublished studies (*d*_+_ = -0.22, *95% CI* = -0.73 to 0.29) and the three adequately powered studies (*d*_+_ = -0.21, *95% CI* = -0.47 to 0.04) of this outcome. The intervention effect on the tendency to make social comparisons appeared resistant to publication and small sample bias, but many of the analyses were not possible due to the small number of tests (*k* = 5).

### Change Techniques and Improvement in Body Image

There was significant heterogeneity in the effects of the interventions on body image, *Q*(1) = 176.26, *p* < 0.001, of a moderate-to-high level [114]. This heterogeneity encourages tests to establish whether particular change techniques were associated with improvements in body image. S3 Table presents the change techniques used in each intervention. Of the 48 intervention techniques identified in our taxonomy, 31 techniques were used in at least four interventions and thus could be included in the analyses. The most commonly used techniques were: discuss the causes of negative body image (*k* = 23), provide physical activity exercises (*k* = 22), discuss cognitions and their role in body image (*k* = 19), teach self-monitoring and restructuring of cognitions (*k* = 17), discuss the consequences of negative body image (*k* = 17), and teach self-monitoring of behaviour (*k* = 17).

Table 5 presents the results of meta-regressions of body image on each of the 31 change techniques (where *k* ≥ 4). Twelve change techniques were significantly associated with larger intervention effects on body image. Interventions were more effective if they discussed cognitions and their role in body image, taught monitoring and restructuring of cognitions, changed negative body language, and incorporated guided imagery, exposure, and size-estimate exercises. Interventions also had a larger effect on body image if they provided relapse-prevention strategies and stress management training, and if they involved discussing the concept of body image, the causes of negative body image, the consequences of negative body image, or the behavioural expression of negative body image. Three of the 31 techniques–providing self-esteem enhancement exercises, discussing individual differences, and discussing physical activity–were associated with *poorer* body image. Although it would have been desirable to undertake multivariate meta-regression analyses (to determine which change techniques best predict improvement when the other techniques are taken into account), the modest number of available tests (*k* = 62) and high correlations between the use of the effective techniques (range = |0.49 to 0.85|, *Mdn* = 0.69, *M* = 0.69) precluded these analyses (for discussion of the “co-occurrence” of behaviour change techniques, see [115]).

**Table 5:** Effect of Specific Change Techniques on Body Image.

Technique		*N*	*k*	β	*SE*	*R* ^2^
*General cognitive-behavioural techniques for improving body image*						
	Discuss cognitions and their role in body image	939.5^a^	19	0.53***	0.13	35.95
	Teach self-monitoring and restructuring of cognitions	823.5^a^	17	0.61***	0.13	40.81
	Teach self-monitoring of behaviour	971	17	0.03	0.15	2.78
	Change negative body language	602	15	0.61***	0.14	45.18
	Change the biased focus toward the body	240	4	-0.18	0.26	2.18
	Conduct guided imagery exercises	359	11	0.38*	0.18	10.22
	Conduct exposure exercises	689	15	0.56***	0.14	28.48
	Write about the body	651	9	0.08	0.18	2.42
	Provide size-estimate exercises	128	6	0.82**	0.27	23.30
	Prompt action-planning	255	7	0.43	0.22	10.21
	Provide relapse-prevention strategies	559	15	0.75***	0.14	58.57
	Provide stress management training	498	14	0.66***	0.15	41.17
*Techniques for enhancing physical fitness*						
	Provide physical activity exercises	1,088	22	-0.01	0.14	2.91
*Techniques providing media-literacy and promoting media resistance*						
	Provide media literacy training	527.5^a^	8	-0.03	0.19	3.24
	Discuss the beauty ideal	734	13	0.06	0.16	1.91
*Techniques designed to enhance self-esteem*						
	Discuss self-esteem	524	6	0.05	0.21	3.15
	Provide self-esteem enhancement exercises	368	4	-0.49*	0.23	8.83
	Discuss individual differences	642	6	-0.59**	0.18	25.79
	Discuss alternatives to focusing on appearance	698	12	-0.15	0.17	0.03
	Discuss interpersonal relations	862	10	-0.32	0.16	8.21
	Teach interpersonal skills	439	6	-0.36	0.20	5.80
	Discuss social comparisons	506	7	0.11	0.20	1.69
	Provide social comparison exercises	560	7	-0.17	0.19	1.04
*Techniques providing psychoeducation related to body image and healthy lifestyle*						
	Discuss the concept of body image	1,069	16	0.32*	0.15	6.68
	Discuss the causes of negative body image	1,494.5^a^	23	0.29*	0.13	10.12
	Discuss the consequences of negative body image	750.5^a^	17	0.47**	0.14	23.32
	Discuss the behavioural expression of negative body image	327	10	0.67**	0.18	30.52
	Discuss healthy eating	512	7	-0.21	0.20	0.26
	Discuss physical activity	871	11	-0.36*	0.16	12.65
	Discuss eating pathology	397	8	0.35	0.20	8.85
*Additional techniques for improving body image*						
	Provide mindfulness exercises	607.5^a^	4	0.24	0.24	0.22

*k* = number of effect sizes; β = beta from metaregression; *SE* = standard error; *R* ^2^ = percentage of variance explained by the change technique.

^a^ A 0.5 results from a study where the sample size for the control condition was halved (to accommodate comparison with two experimental conditions/interventions) and where the change technique was used in one intervention but not the other.

* *p* < 0.05

** *p* < 0.01

*** *p* < 0.001.

### Moderation by Features of the Sample, Intervention, and Methodology

S3 Table shows the moderator features for each intervention separately. The majority of interventions targeted samples that were not screened for having a negative body image (*k* = 40), and samples at early adulthood (*k* = 34). Most interventions were conducted in a group format (*k* = 39), with a facilitator present (*k* = 44), and involved multiple sessions (*k* = 48). Interventions were most often compared to a passive control group (*k* = 35) and included only a pretest and immediate posttest measurement (*k* = 44).

Table 6 presents findings for meta-regression of effect sizes on features of the sample, intervention, and methodology. Interventions that selected participants for the presence of a negative body image produced significantly larger improvements in body image (*d*_+_ = 0.79) compared to interventions where participants were not screened for having a negative body image (*d*_+_ = 0.14), *Q*(1) = 81.16, *p* < 0.001. The percentage of females in the sample did not moderate the effect of the interventions on body image, β = 0.001, *SE* = 0.002, *p* = 0.42. Interventions targeting participants in adolescence showed significantly larger improvements in body image (*d*_+_ = 0.79) compared to interventions targeting participants at childhood (*d*_+_ = -0.03), *Q*(1) = 29.30, *p* < 0.001, and early adulthood (*d*_+_ = 0.33), *Q*(1) = 9.86, *p* < 0.001. The effect size for interventions targeted at children was not reliable (*95% CI* = -0.16 to 0.10). Interventions targeting participants in early adulthood showed significantly larger improvements in body image compared to interventions targeting participants in childhood, *Q*(1) = 18.85, *p* < 0.001, but significantly smaller improvements compared to interventions targeting participants in middle adulthood, *Q*(1) = 21.01, *p* < 0.001. The effects were larger for participants in middle adulthood (*d*_+_ = 0.70) compared to childhood, *Q*(1) = 65.95, *p* < 0.001, but did not differ compared to adolescence (*p* = 0.36). None of the interventions targeted participants at late adulthood.

**Table 6:** Moderators of Intervention Effects on Body Image.

Moderator		*N*	*k*	*d*_+_ (95% CI)	*Q*	*I* ^2^
Sample						
	Selected	1,148	22	0.79 (0.53 to 1.05)	83.77***	74.9
	Nonselected	2,698	40	0.14 (0.06 to 0.22)	40.04	2.6
Age						
	Childhood	938	13	-0.03 (-0.16 to 0.10)	11.91	0
	Adolescence	232	5	0.79 (0.16 to 1.42)	22.31***	82.1
	Early adulthood	1,549	34	0.33 (0.22 to 0.44)	36.08	8.5
	Middle adulthood	1,127	10	0.70 (0.34 to 1.06)	71.08***	87.3
Intervention format						
	Group	1,968	39	0.50 (0.32 to 0.69)	137.29***	72.3
	Individual	1,878	23	.20 (0.09 to 0.31)	29.39	25.2
Presence of facilitator						
	Facilitator present	2,143	44	0.49 (0.33 to 0.66)	146.68***	70.7
	No facilitator present	1,703	18	0.16 (0.06 to 0.27)	18.41	7.6
Number of sessions						
	Single-session	749	14	0.18 (0.03 to 0.32)	13.11	0.8
	Multisession	3,097	48	0.45 (0.31 to 0.60)	160.58***	70.7
Type of control group						
	Active	1,544	27	0.27 (0.11 to 0.44)	61.17***	57.5
	Passive	2,302	35	0.47 (0.30 to 0.63)	108.54***	68.7
Time to follow-up						
	Posttest only	2,530	44	0.46 (0.31 to 0.62)	134.56***	68.0
	Short-term	1,316	18	0.19 (0.03 to 0.36)	32.28*	47.3

*k* = number of effect sizes; *d*_*+*_ = sample-weighted average effect size; *95% CI* = 95% confidence interval; *Q* = homogeneity *Q* statistic; *I* ^*2*^ = homogeneity *I* ^*2*^ statistic.

* *p* < 0.05

*** *p* < 0.001.

Interventions delivered in a group format resulted in significantly greater improvements in body image (*d*_+_ = 0.50) compared to interventions delivered on an individual basis (*d*_+_ = 0.20), *Q*(1) = 21.15, *p* < 0.001. Interventions where a facilitator was present (*d*_+_ = 0.49) were significantly more effective than were interventions where no facilitator was present (*d*_+_ = 0.16), *Q*(1) = 25.54, *p* < 0.001. Multisession interventions also produced significantly larger improvements in body image (*d*_+_ = 0.45) compared to single-session interventions (*d*_+_ = 0.18), *Q*(1) = 11.33, *p* = 0.001. Interventions tested against an active control group reported significantly smaller improvements in body image (*d*_+_ = 0.27) compared to interventions tested against a passive control group (*d*_+_ = 0.47), *Q*(1) = 8.45, *p* = 0.004. The intervention effect was significantly larger for studies with an immediate posttest (*d*_+_ = 0.47) compared to studies with a short-term follow-up (*d*_+_ = 0.19), *Q*(1) = 15.98, *p* < 0.001. None of the interventions included a longer-term follow-up.

## Discussion

The aim of this meta-analysis was to determine the effectiveness of stand-alone interventions to improve body image and to identify the specific change techniques that are associated with improvement. Overall, the effect size for improvement in body image was reliable and of small-to-medium magnitude. However, the effect size for studies with high risk of bias was significantly larger than the effect size for less biased studies, where a small effect was observed. Moreover, correction for publication bias and small sample bias also indicated that the effect of interventions on body image was of small magnitude. In sum, the present findings suggest that the overall effect of stand-alone interventions on body image is inflated by biases both within and across studies. After correcting for bias, interventions are found to generate a small, but reliable, improvement in body image. With regard to the secondary outcomes, the overall analyses indicated that interventions produced a reliable and small-to-medium effect on internalisation of the beauty ideal and a large effect on the tendency to make social comparisons. However, the effects for these outcomes were small–and no longer reliable–once corrections for publication bias and small sample bias had been applied. Thus, whereas previous reviews of interventions in this area indicate that sample-weighted average effect sizes ranged from small to large, the present meta-analysis finds that stand-alone interventions have a small effect on body image, and negligible effects on beauty ideal internalisation and social comparison tendencies.

### Which Change Techniques Were Effective at Improving Body Image?

A novel feature of our review is that interventions were coded and evaluated at the technique level and not merely at the level of the broad approach taken. So doing afforded the opportunity to identify which specific change techniques are associated with improvements in body image, in an equivalent manner to the procedures that are well established in research on behaviour change [75]. Of the 48 change techniques that we defined, 31 techniques were used in at least four interventions and could be analysed via meta-regression [75]. Twelve change techniques were associated with significant improvements in body image. These techniques included discussing the role of cognitions in body image, and teaching monitoring and restructuring of cognitions. Cognitive distortions related to body image–such as dichotomous thinking [116] or overestimation of negative social feedback about one’s body [117]–create distress, and serve to reinforce and maintain negative body image [118]. Exercises that train participants to monitor and restructure their cognitions may make them aware of the complex interplay between their thoughts, feelings, and behaviour, thereby helping them to break this negative cycle [24,119,120]. Cognitive restructuring may also help people to approach day-to-day situations in more adaptive ways, for example by using positive self-talk before a social gathering to remind oneself that appearance does not determine self-worth [119].

Changing negative body language also improved body image. This technique directly targets the language that people use to describe or talk about their body, with the aim of helping individuals to use objective or positive terms rather than negative, judgmental language. For instance, *fat talk* involves comments or conversations that are focussed on weight and appearance, and are typically evaluative and judgemental (e.g., “I’m so fat!” or, “I should skip meals to help me lose weight,”[121], p.173). Engaging in fat talk is related to negative body image and greater levels of psychological distress, and affects body image above and beyond merely thinking negatively about one’s body [121–123]. The current findings underline the need to address such harmful self-talk in order to improve body image.

Guided imagery, exposure exercises, and size-estimate exercises all emerged as effective techniques to improve body image. Guided imagery and exposure exercises are targeted at experiential and behavioural avoidance, which perpetuate negative body image [24]. Exposure exercises may be effective because they create “heart level” emotional beliefs. That is, positive thoughts about one’s body that are accompanied by the feeling that the respective thoughts are true and convincing, and are experienced as more than mere dispassionate thinking [119,124]. According to Bennett-Levy [119], exposure exercises are one of the most direct methods for challenging maladaptive thinking, and for testing and improving the believability of new, adaptive thoughts. Size-estimate exercises may operate in similar fashion, as they require participants to estimate the size of a body part and then to objectively measure that body part. The present findings suggest that it may be important for interventions to include such exercises, notwithstanding any reservations that participants or intervention practitioners may have (e.g., that these techniques are anxiety-provoking, [24]).

Two techniques from Abraham and Michies’ [42] taxonomy of behaviour change techniques–stress management training and relapse prevention–were associated with improved body image. These findings would seem to speak to the importance of learning adaptive coping strategies to deal with challenges and setbacks in efforts to enhance body image. A further four effective techniques involved psychoeducation. Although psychoeducation has been associated with smaller effect sizes in interventions targeting other issues (e.g., programs to prevent eating disorders or reduce alcohol consumption, [125,126]), teaching participants about the concept of body image and its causes and consequences, as well as how it is expressed behaviourally, was associated with improved body image here. These findings are not consistent with the idea that psychoeducation may actually instil negative body image (e.g., by glamorising eating pathology, [30]). Psychoeducation may give people a better understanding of the factors that precipitate and exacerbate negative body image, and may help them to recognise and manage the impact of ‘triggers’ (e.g., reading fashion magazines).

Three change techniques were contra-indicated in the present review: Providing self-esteem enhancement exercises, discussing physical fitness, and discussing individual differences each decreased the effectiveness of the interventions. Findings regarding self-esteem enhancement exercises should be interpreted with caution, however, because the four tests that incorporated this technique are derived from the same study [127], and additional tests are needed. One explanation for the negative effect of discussing physical fitness is that discussing physical activity may inadvertently draw attention to weight and appearance, and highlight societal standards for physical fitness and attractiveness [30]. Along the same lines, discussing individual differences could underscore the discrepancy between an individual’s current body and the ‘ideal body.’ Similar reasoning could explain why providing media literacy did not improve body image. Although a wealth of evidence points to the adverse impact of the media on body image [27,28], and media literacy may increase media scepticism, such increased scepticism may not be sufficient to improve body image [29]. It is possible that scepticism occurs at the level of reasoning and logic (e.g., knowing that the beauty ideal is unachievable) but does not get translated into “heart level” emotional beliefs [124]. Perceived self-efficacy may also play a role as people may not feel confident in their ability to control media influences on their body image. Future research might usefully measure putative moderators (e.g., scepticism, perceived self-efficacy) in order to clarify whether media literacy and media resistance interventions are effective in certain circumstances.

### The Influence of Features of the Sample, Intervention, and Methodology on Intervention Effectiveness

Mirroring findings for other types of interventions (e.g., aimed at preventing depression or eating disorders, [126,128]), selected body image interventions were more effective than nonselected ones. In addition, interventions were more effective when they targeted participants at adolescence or middle adulthood, when they were delivered in multiple sessions, in a group format, with a facilitator present, and when the intervention was tested against a passive control group and included only an immediate posttest measurement. These findings raise three issues. First, it is noteworthy that 10 of the interventions (16%) were targeted at participants at middle adulthood and that these interventions had large effects on body image. Similar to adolescence–where interventions produced the largest effects on body image–the period of middle adulthood may be a time when individuals are particularly vulnerable to developing a negative body image (e.g., due to menopause or changes in body fat-to-muscle composition, [129,130]). The present findings highlight the potential for intervention in participants at middle adulthood, and indicate that additional research about body image in people at middle adulthood is important. Second, although interventions targeting body image had smaller effects for participants that were not screened for having a negative body image and for participants at childhood, it is possible that interventions could buffer against future challenges and help to prevent the development of negative body image over time. Future studies could carefully consider the appropriate age at which to target participants, and include long-term follow-ups to test whether control participants develop a more negative body image compared to participants who receive the intervention. Third, the benefit of multisession interventions will need to be weighed against the potential costs (e.g., the resources needed for delivery, [40]). It may be important for future studies to investigate efficient ways to administer multisession interventions, or to strengthen extant single-session interventions.

### Limitations and Directions for Future Research

The current meta-analytic review is limited by biases both within and across studies. None of the individual studies could be coded as low risk, and the majority were considered high risk with regard to blinding of participants and outcome assessment, which can inflate estimates of intervention effects especially on subjective outcomes [131,132]. Approximately one-third of the studies exhibited unclear risk of bias in summary analyses because insufficient information was provided in the primary reports. None of the studies provided sufficient information about selective reporting of outcomes; this is problematic because reporting bias (e.g., failure to report nonsignificant effects on particular outcomes) has considerable influence on research findings [49]. Regarding bias across studies, the trim and fill analyses imputed 21 additional effect sizes; this value amounts to one-third of the total number of tests (*k* = 62). It appears that a considerable proportion of interventions that observed negative or null effects on body image either were not submitted or were not published. Interventions involving small samples (*n* < 35 per cell) were also commonplace, and only one-quarter of the interventions had at least 55% power to detect a medium-sized effect. Equivalent problems were observed with the secondary outcomes that appeared to be reliable in the overall analyses.

Coyne et al. [54], Ferguson and Brannick [133], and Ioannidis, Munafò, Fusar-Poli, Nosek, and David [134] all offered helpful recommendations for tackling bias. First, risk of bias within individual studies and across studies should routinely be tested in future meta-analyses. Second, appropriate procedures to correct for these sources of bias should be undertaken. These procedures include extensive searches for unpublished studies and the use of trim and fill, Coyne et al.’s [54] computation, or similar statistical techniques. Third, the use of study registries (e.g., http://clinical-trials.gov) and registries that allow researchers to pre-specify design and analysis plans (e.g., Open Science Framework; http://osf/.io) would enable meta-analysts both to discover studies that were conducted but were not reported, and to identify instances of selective reporting, and could make the need for statistical post-hoc methods for assessing publication bias obsolete [135]. Researchers should aim to conduct interventions with sufficiently large sample sizes, and follow established reporting guidelines (e.g., the CONSORT Statement, [136]) to provide readers and meta-analysts with complete and transparent information about the methodology and findings of the research.

The present findings suggest several considerations that will be important in future stand-alone interventions to improve body image. The majority of the studies reviewed here recruited female participants in their early adulthood, and tested intervention effects against a passive control group, using outcomes measured in the immediate wake of the intervention. Active control groups provide a stricter test of intervention effects than do passive control groups, and the present findings–like previous reviews [137]–indicate that the use of passive control conditions is associated with larger intervention effect sizes. The present findings also showed that intervention effects diminished over time, and none of the studies followed participants for longer than 3 months. Future studies should therefore prioritise active control conditions and longer-term follow-ups and test stand-alone interventions among under-represented samples (e.g., men, adolescents).

### Conclusions

The present meta-analysis addressed two questions: How effective are stand-alone interventions at improving body image, and what change techniques lead to improvements in body image? The answer to the first question is that improvement in body image attributable to stand-alone interventions is small in magnitude, after correcting for publication and small sample bias. Stand-alone interventions have negligible effects on internalisation of the beauty ideal and social comparison tendencies. To answer the second question, a novel and reliable taxonomy of change techniques was developed. Three techniques were contra-indicated whereas 12 techniques were associated with improved body image. The present findings suggest that more, better powered, and higher quality interventions to improve body image are needed and that increased efforts to combat publication bias are warranted. The findings also specify several effective change techniques that can and should be tested in future research.

## Supporting Information

S1 File List of Studies Included in the Meta-Analysis. (DOCX)

S2 File Dataset for the Meta-Analytic Review of Stand-Alone Interventions to Improve Body Image. (XLSX)

S3 File Datasheet for Risk of Bias Within Studies as Coded Using the Cochrane Collaboration’s Tool for Assessing Risk of Bias. (XLSX)

S1 Table Completed PRISMA Checklist. (DOC)

S2 Table Risk of Bias Within Individual Studies. (DOCX)

S3 Table Change Techniques Deployed and Moderator Features for Each Intervention. (DOCX)
