# Supplementary material for: A Meta-Analytic Review of Stand-Alone Interventions to Improve Body Image
Source: PLoS One. 2015 Sep 29;10(9):e0139177. doi: 10.1371/journal.pone.0139177 (PMC4587797; doi:10.1371/journal.pone.0139177)
Supplement: S1 File — (DOCX) [file pone.0139177.s001.docx]

**S1 File. List of Studies Included in the Meta-Analysis**

1. Albertson ER, Neff KD, Dill-Shackleford KE. Self-Compassion and Body Dissatisfaction in Women: A Randomized Controlled Trial of a Brief Meditation Intervention. Mindfulness. 2014;1-11.

2. Alleva JM, Martijn C, Jansen A, Nederkoorn C. Body Language: Affecting Body Satisfaction by Describing the Body in Functionality Terms. Psychol Women Q. 2013;38(2):181–96.

3. Alleva JM, Martijn C, Van Breukelen GJP, Jansen A, Karos K. *Expand Your Horizon*: A programme that improves body image and reduces self-objectification by training women to focus on body functionality. Forthcoming 2015.

4. Arbour KP, Martin Ginis KA. Improving body image one step at a time: Greater pedometer step counts produce greater body image improvements. Body Image. 2008;5(4):331–6.

5. Aşçi FH. The effects of physical fitness training on trait anxiety and physical self-concept of female university students. Psychol Sport Exerc. 2003;4(3):255–64.

6. Aşçi FH, Kin A, Kosar S. Effect of participation in an 8 week aerobic dance and step aerobics program on physical self-perception and body image satisfaction. Int J Sport Psychol. 1998;29(4):366–75.

7. Aşçi FH. The effects of step dance on physical self-perception of female and male university students. Int J Sport Psychol. 2002;33(4):431–42.

8. Bhatnagar KAC. Effectivness and feasibility of a cognitive-behavioral group intervention for body image disturbance in women with eating disorders [dissertation]. Cleveland (OH): Case Western Reserve University; 2013.

9. Burgess G, Grogan S, Burwitz L. Effects of a 6-week aerobic dance intervention on body image and physical self-perceptions in adolescent girls. Body Image. 2006;3(1):57–66.

10. Butters JW, Cash TF. Cognitive-behavioral treatment of women’s body-image dissatisfaction. J Consult Clin Psychol. 1987;55(6):889–97.

11. Corning AF, Gondoli DM, Bucchianeri MM, Salafia EHB. Preventing the development of body issues in adolescent girls through intervention with their mothers. Body Image. 2010;7(4):289–95.

12. Cousineau TM, Franko DL, Trant M, Rancourt D, Ainscough J, Chaudhuri A, et al. Teaching adolescents about changing bodies: Randomized controlled trial of an Internet puberty education and body dissatisfaction prevention program. Body Image. 2010;7(4):296–300.

13. Cruz-Ferreira A, Fernandes J, Gomes D, Bernardo LM, Kirkcaldy BD, Barbosa TM, et al. Effects of Pilates-based exercise on life satisfaction, physical self-concept and health status in adult women. Women Health. 2011;51(3):240–55.

14. Delinsky SS, Wilson GT. Mirror exposure for the treatment of body image disturbance. Int J Eat Disord. 2006;39(2):108–16.

15. Divsalar D. Body image dissatisfaction: An intervention study with college women [dissertation]. San Diego (CA): Alliant International University; 2006.

16. Dohnt HK, Tiggemann M. Promoting positive body image in young girls: An evaluation of “Shapesville.” Eur Eat Disord Rev. 2008;16(3):222–33.

17. Duncan MJ, Al-Nakeeb Y, Nevill AM. Effects of a 6-week circuit training intervention on body esteem and body mass index in British primary school children. Body Image. 2009;6(3):216–20.

18. Dunigan BJ, King TK, Morse BJ. A preliminary examination of the effect of massage on state body image. Body Image. 2011;8(4):411–4.

19. Earnhardt JL, Martz DM, Ballard ME, Curtin L. A writing intervention for negative body image. J College Stud Psychother. 2002;17(1):19–35.

20. Emerson EN. The efficacy of a self-administered cognitive behavioural treatment program for body image dissatisfaction in women with subclinical bulimia nervosa [dissertation]. Logan (UT): Utah State University; 1996.

21. Fisher E, Thompson JK. A comparative evaluation of cognitive-behavioral therapy (CBT) versus exercise therapy (ET) for the treatment of body image disturbance. Preliminary findings. Behav Modif. 1994;18(2):171–85.

22. Gehrman CA, Hovell MF, Sallis JF, Keating K. The effects of a physical activity and nutrition intervention on body dissatisfaction, drive for thinness, and weight concerns in pre-adolescents. Body Image. 2006;3(4):345–51.

23. Geraghty AWA, Wood AM, Hyland ME. Attrition from self-directed interventions: Investigating the relationship between psychological predictors, intervention content and dropout from a body dissatisfaction intervention. Soc Sci Med. 2010;71(1):30–7.

24. Grasso K. An expressive writing intervention for body image: A randomized controlled trial [dissertation]. Norfolk (VA): Old Dominion University; 2007.

25. Heinicke BE, Paxton SJ, McLean SA, Wertheim EH. Internet-delivered targeted group intervention for body dissatisfaction and disordered eating in adolescent girls: A randomized controlled trial. J Abnorm Child Psychol. 2007;35(3):379–91.

26. Jansen A, Bollen D, Tuschen-Caffier B, Roefs A, Tanghe A, Braet C. Mirror exposure reduces body dissatisfaction and anxiety in obese adolescents: A pilot study. Appetite. 2008;51(1):214–7.

27. Lew AM, Mann T, Myers H, Taylor S, Bower J. Thin-ideal media and women’s body dissatisfaction: Prevention using downward social comparisons on non-appearance dimensions. Sex Roles. 2007;57(7-8):543–56.

28. Lindwall M, Lindgren EC. The effects of a 6-month exercise intervention programme on physical self-perceptions and social physique anxiety in non-physically active adolescent Swedish girls. Psychol Sport Exerc. 2005;6(6):643–58.

29. Martijn C, Sheeran P, Wesseldijk LW, Merrick H, Webb TL, Roefs A, et al. Evaluative conditioning makes slim models less desirable as standards for comparison and increases body satisfaction. Heal Psychol. 2012;(2008):1–7.

30. Martijn C, Vanderlinden M, Roefs A, Huijding J, Jansen A. Increasing body satisfaction of body concerned women through evaluative conditioning using social stimuli. Heal Psychol. 2010;29(5):514–20.

31. McCabe MP, Ricciardelli LA, Salmon J. Evaluation of a prevention program to address body focus and negative affect among children. J Health Psychol. 2006;11(4):589–98.

32. McLean SA, Paxton SJ, Wertheim EH. A body image and disordered eating intervention for women in midlife: A randomized controlled trial. J Consult Clin Psychol. 2011;79(6):751–8.

33. Murphy K. The efficacy of a cognitive-behavioral group treatment of body image disturbances in females with low and high levels of bulimic symptoms [dissertation]. Binghampton (NY): State University of New York; 1994.

34. Özdemir RA, Çelik Ö, Aşçı FH. Exercise interventions and their effects on physical self-perceptions of male university students. Int J Psychol. 2010;45(3):174–81.

35. Paxton SJ, McLean SA, Gollings EK, Faulkner C, Wertheim EH. Comparison of face-to-face and internet interventions for body image and eating problems in adult women: An RCT. Int J Eat Disord. 2007;40(8):692–704.

36. Pearson AN, Follette VM, Hayes SC. A pilot study of Acceptance and Commitment Therapy as a workshop intervention for body dissatisfaction and disordered eating attitudes. Cogn Behav Pract. 2012;19(1):181–97.

37. Peterson RD, Tantleff-Dunn S, Bedwell JS. The effects of exposure to feminist ideology on women’s body image. Body Image. 2006;3(3):237–46.

38. Ridolfi DR, Vander Wal JS. Eating disorders awareness week: the effectiveness of a one-time body image dissatisfaction prevention session. Eat Disord. 2008;16(5):428–43.

39. Rosen JC, Reiter J, Orosan P. Cognitive-behavioral body image therapy for body dysmorphic disorder. J Consult Clin Psychol. 1995;63(2):263–9.

40. Rosen JC, Saltzberg E, Srebnik D. Cognitive behavior therapy for negative body image. Behav Ther. 1989;20(3):393–404.

41. Rosen J, Orosan P, Reiter J. Cognitive behavior therapy for negative body image in obese women. Behav Ther. 1996;26:25–42.

42. Stanford JN, McCabe MP. Evaluation of a body image prevention programme for adolescent boys. Eur Eat Disord Rev. 2005;13(5):360–70.

43. Waggoner IRM. Cognitive-behavior therapy and cognitive therapy for body image awareness in sixth grade females [dissertation]. Auburn (AL): Auburn University; 1999.
