# Supplementary material for: A Meta-Analytic Review of Stand-Alone Interventions to Improve Body Image
Source: PLoS One. 2015 Sep 29;10(9):e0139177. doi: 10.1371/journal.pone.0139177 (PMC4587797; doi:10.1371/journal.pone.0139177)
Supplement: S2 Table — (DOCX) [file pone.0139177.s005.docx]

**S2 Table. Risk of Bias Within Individual Studies***.*

| **Study** | **Summary assessment** | **Random sequence generation** | **Allocation concealment** | **Blinding of participants** | **Blinding of outcome assessment** | **Incomplete outcome data** | **Selective reporting** | **Other bias** |
| --- | --- | --- | --- | --- | --- | --- | --- | --- |
| Albertson et al. (2014) | High | Unclear | Unclear | High | High | High | Unclear | Low |
| Alleva et al. (2014) – Study 1^a^ | Unclear | Low | Unclear | Low | Low | Low | Unclear | Low |
| Alleva et al. (2014) – Study 1^b^ | Unclear | Low | Unclear | Low | Low | Low | Unclear | Low |
| Alleva et al. (2014) – Study 2 | Unclear | Low | Unclear | Low | Low | Low | Unclear | Low |
| Alleva et al. (2015) | Unclear | Low | Unclear | Low | Low | Low | Unclear | Low |
| Arbour & Ginis (2008) | High | Unclear | Unclear | Low | Low | High | Unclear | Unclear |
| Asci (2002)^a^ | Unclear | Unclear | Unclear | Low | Low | Low | Unclear | Low |
| Asci (2002)^b^ | High | Unclear | Unclear | Low | Low | High | Unclear | Low |
| Asci (2003) | Unclear | Unclear | Unclear | Low | Low | Low | Unclear | Low |
| Asci et al. (1998)^c^ | High | Unclear | Unclear | Low | High | Low | Unclear | Low |
| Asci et al. (1998)^d^ | High | Unclear | Unclear | Low | High | Low | Unclear | Low |
| Bhatnagar (2013) | High | Unclear | Unclear | High | High | High | Unclear | Low |
| Burgess et al. (2006) | Unclear | Unclear | Unclear | Low | Low | Low | Unclear | Low |
| Butters & Cash (1987) | High | Low | Low | High | High | Low | Unclear | Low |
| Corning et al. (2010) | High | Low | Low | High | High | Low | Unclear | Unclear |
| Cousineau et al. (2010) | Unclear | Low | Unclear | Low | Low | Low | Unclear | Low |
| Cruz-Ferreira et al. (2011) | High | Low | Low | High | High | High | Unclear | Low |
| Delinsky & Wilson (2006) | Unclear | Unclear | Unclear | Low | Low | Low | Unclear | Low |
| Divsalar (2006)^e^ | High | Low | Low | High | High | Low | Unclear | Low |
| Divsalar (2006)^f^ | High | Low | Low | High | High | Low | Unclear | Low |
| Dohnt & Tiggemann (2008) | Unclear | Unclear | Unclear | Low | Low | Low | Unclear | Low |
| Duncan et al. (2009)^b^ | High | Unclear | Unclear | Low | High | Low | Unclear | Low |
| Duncan et al. (2009)^a^ | High | Unclear | Unclear | Low | High | Low | Unclear | Low |
| Dunigan et al. (2011) | Unclear | Unclear | Low | Low | Low | Low | Unclear | Low |
| Earnhardt et al. (2002) | High | High | High | Low | Low | High | Unclear | Low |
| Emerson (1995) | High | Unclear | Unclear | High | High | High | Unclear | Low |
| Fisher & Thompson (1994)^g^ | High | Unclear | Unclear | High | High | Low | Unclear | Low |
| Fisher & Thompson (1994)^h^ | High | Unclear | Unclear | High | High | Low | Unclear | Low |
| Gehrman et al. (2006)^a^ | Unclear | Unclear | Unclear | Low | Low | Low | Unclear | Low |
| Gehrman et al. (2006)^b^ | High | Unclear | Unclear | Low | Low | Low | Unclear | High |
| Geraghty et al. (2010)^i^ | High | Low | Low | High | High | High | Unclear | Low |
| Geraghty et al. (2010)^j^ | High | Low | Low | High | High | High | Unclear | Low |
| Grasso (2007) | High | High | High | Low | Low | High | Unclear | High |
| Heinicke et al. (2007) | High | Low | Low | High | High | High | Unclear | Low |
| Jansen et al. (2008) | High | Unclear | Unclear | High | High | Low | Unclear | Unclear |
| Lew et al. (2007) | Unclear | Unclear | Unclear | Low | Low | Unclear | Unclear | Low |
| Lindwall & Lindgren (2005) | High | Low | Low | High | High | High | Unclear | Low |
| Martijn et al. (2012) - Study 2 | Unclear | Unclear | Unclear | Low | Low | Low | Unclear | Low |
| Martijn et al. (2010)^k^ | Unclear | Unclear | Unclear | Low | Low | Unclear | Unclear | Low |
| Martijn et al. (2010)^l^ | Unclear | Unclear | Unclear | Low | Low | Unclear | Unclear | Low |
| McCabe et al. (2006)^a, m^ | High | Unclear | Unclear | High | High | Low | Unclear | Unclear |
| McCabe et al. (2006)^a, n^ | High | Unclear | Unclear | High | High | Low | Unclear | Unclear |
| McCabe et al. (2006)^b, m^ | High | Unclear | Unclear | High | High | Low | Unclear | Unclear |
| McCabe et al. (2006)^b, n^ | High | Unclear | Unclear | High | High | Low | Unclear | Unclear |
| McLean et al. (2011) | High | Unclear | Unclear | High | High | High | Unclear | Low |
| Murphy (1994)^k^ | High | Unclear | Unclear | High | High | Low | Unclear | Low |
| Murphy (1994)^l^ | High | Unclear | Unclear | High | High | Low | Unclear | Low |
| Özdemir et al. (2010)^o^ | High | Unclear | Unclear | High | High | Low | Unclear | Low |
| Özdemir et al. (2010)^p^ | High | Unclear | Unclear | High | High | Low | Unclear | Low |
| Özdemir et al. (2010)^q^ | High | Unclear | Unclear | High | High | Low | Unclear | Low |
| Paxton et al. (2007)^r^ | High | Low | Low | High | High | High | Unclear | Low |
| Paxton et al. (2007)^s^ | High | Low | Low | High | High | High | Unclear | Low |
| Pearson et al. (2012) | High | Low | Low | High | High | High | Unclear | Low |
| Peterson et al. (2006)^t^ | Unclear | Unclear | Unclear | Low | Low | Low | Unclear | Low |
| Peterson et al. (2006)^u^ | Unclear | Unclear | Unclear | Low | Low | Low | Unclear | Low |
| Ridolfi & Vander Wal (2008) | High | Unclear | Unclear | High | High | Low | Unclear | Unclear |
| Rosen et al. (1995)^v^ | High | Unclear | Unclear | High | High | Low | Unclear | Low |
| Rosen et al. (1995)^w^ | High | Unclear | Unclear | High | High | Low | Unclear | Low |
| Rosen et al. (1989) | Unclear | Unclear | Unclear | Low | Low | Low | Unclear | Unclear |
| Stanford & McCabe (2005) | Unclear | Unclear | Unclear | Unclear | Unclear | Unclear | Unclear | Unclear |
| Waggoner (1999)^g^ | Unclear | Unclear | Unclear | Unclear | Unclear | Low | Unclear | Unclear |
| Waggoner (1999)^x^ | Unclear | Unclear | Unclear | Unclear | Unclear | Low | Unclear | Unclear |

^a^ Females. ^b^ Males. ^c^ Dance aerobics. ^d^ Step aerobics. ^e^ Video Intervention 1. ^f^ Video Intervention 2. ^g^ Cognitive-behavioural therapy (CBT). ^h^ Fitness training intervention. ^i^ Gratitude diaries. ^j^ Monitoring and restructuring. ^k^ High-risk women. ^l^ Low-risk women. ^m^ 3^rd^ and 4^th^ grade students. ^n^ 5^th^ and 6^th^ grade students. ^o^ Cycling. ^p^ Running. ^q^ Swimming. ^r^ Face-to-face intervention. ^s^ Internet intervention. ^t^ Feminist intervention. ^u^ Psychoeducation intervention. ^v^ Rosen, Orosan, & Reiter [76]. ^w^ Rosen, Reiter, & Orosan [77]. ^x^ Cognitive therapy. Risk of bias within individual studies was assessed using the Cochrane Collaboration’s Tool for Assessing Risk of Bias [50].
