# Supplementary material for: A Meta-Analytic Review of Stand-Alone Interventions to Improve Body Image
Source: PLoS One. 2015 Sep 29;10(9):e0139177. doi: 10.1371/journal.pone.0139177 (PMC4587797; doi:10.1371/journal.pone.0139177)
Supplement: S3 Table — (DOCX) [file pone.0139177.s006.docx]

**S3 Table. Change Techniques Deployed and Moderator Features for Each Intervention.**

| **Study** | **Change techniques** | **Sample** | **Age** | **Gender** | **Intervention format** | **Presence of facilitator** | **Number of sessions** | **Type of control** | **Follow-up** |
| --- | --- | --- | --- | --- | --- | --- | --- | --- | --- |
| Albertson et al. (2014) | 48 | Selected | Middle adulthood | 100 | Individual | No | Multisession | Passive | Short-term |
| Alleva et al. (2014) – Study 1^a^ | 5, 8 | Nonselected | Early adulthood | 100 | Individual | No | Single-session | Active | Short-term |
| Alleva et al. (2014) – Study 1^b^ | 5, 8 | Nonselected | Early adulthood | 0 | Individual | No | Single-session | Active | Short-term |
| Alleva et al. (2014) – Study 2 | 5, 8 | Nonselected | Middle adulthood | 100 | Individual | No | Single-session | Active | Short-term |
| Alleva et al. (2015) | 5, 8, 29 | Selected | Early adulthood | 100 | Individual | No | Multisession | Active | Short-term |
| Arbour & Ginis (2008) | 3, 10, 11, 13, 20 | Nonselected | Middle adulthood | 100 | Group | No | Multisession | Active | Posttest only |
| Asci (2002)^a^ | 20 | Nonselected | Early adulthood | 100 | Group | Yes | Multisession | Active | Posttest only |
| Asci (2002)^b^ | 20 | Nonselected | Early adulthood | 0 | Group | Yes | Multisession | Active | Posttest only |
| Asci (2003) | 20 | Nonselected | Early adulthood | 100 | Group | Yes | Multisession | Active | Posttest only |
| Asci et al. (1998)^c^ | 20 | Nonselected | Early adulthood | 100 | Group | Yes | Multisession | Active | Posttest only |
| Asci et al. (1998)^d^ | 20 | Nonselected | Early adulthood | 100 | Group | Yes | Multisession | Active | Posttest only |
| Bhatnagar (2013) | 1, 2, 4, 6, 7, 10, 17, 18, 37, 38, 39, 40, 43 | Selected | Early adulthood | 100 | Group | Yes | Multisession | Passive | Posttest only |
| Burgess et al. (2006) | 20 | Selected | Adolescence | 100 | Group | Yes | Multisession | Active | Posttest only |
| Butters & Cash (1987) | 1, 2, 3, 6, 7, 17, 18, 29, 38, 39 | Selected | Early adulthood | 100 | Individual | Yes | Multisession | Passive | Posttest only |
| Corning et al. (2010) | 2, 3, 4, 7, 10, 14, 15, 16, 21, 22, 23, 29, 32, 33, 38, 41 | Nonselected | Adolescence | 100 | Individual | Yes | Multisession | Passive | Short-term |
| Cousineau et al. (2010) | 26, 28, 31, 32, 34, 41, 42 | Nonselected | Childhood | 57 | Individual | No | Multisession | Active | Short-term |
| Cruz-Ferreira et al. (2011) | 20 | Nonselected | Middle adulthood | 100 | Individual | Yes | Multisession | Passive | Posttest only |
| Delinsky & Wilson (2006) | 1, 3, 4, 7, 12, 14, 15, 38, 47 | Selected | Early adulthood | 100 | Individual | Yes | Multisession | Active | Short-term |
| Divsalar (2006)^e^ | 21, 22, 23, 30, 34, 36 | Selected | Early adulthood | 100 | Group | No | Single-session | Passive | Posttest only |
| Divsalar (2006)^f^ | 1, 21, 22, 34, 36, 38, 39 | Selected | Early adulthood | 100 | Group | No | Single-session | Passive | Posttest only |
| Dohnt & Tiggemann (2008) | 22, 26, 28, 30, 32, 37, 41, 42 | Nonselected | Childhood | 100 | Group | Yes | Single-session | Active | Short-term |
| Duncan et al. (2009)^b^ | 20 | Nonselected | Childhood | 0 | Group | Yes | Multisession | Passive | Short-term |
| Duncan et al. (2009)^a^ | 20 | Nonselected | Childhood | 100 | Group | Yes | Multisession | Passive | Short-term |
| Dunigan et al. (2011) | 49 (Massage) | Nonselected | Early adulthood | 100 | Individual | Yes | Single-session | Active | Posttest only |
| Earnhardt et al. (2002) | 6, 8, 38 | Nonselected | Early adulthood | 100 | Individual | Yes | Multisession | Active | Short-term |
| Emerson (1995) | 1, 2, 3, 6, 7, 17, 18, 29, 33, 38, 39,40 | Selected | Early adulthood | 100 | Individual | No | Multisession | Passive | Posttest only |
| Fisher & Thompson (1994)^g^ | 1, 2, 3, 6, 7, 17, 18, 29, 38, 39 | Selected | Early adulthood | 100 | Group | Yes | Multisession | Passive | Posttest only |
| Fisher & Thompson (1994)^h^ | 20, 37, 42 | Selected | Early adulthood | 100 | Group | Yes | Multisession | Passive | Posttest only |
| Gehrman et al. (2006)^a^ | 3, 20, 41, 42 | Nonselected | Childhood | 100 | Group | Yes | Multisession | Active | Posttest only |
| Gehrman et al. (2006)^b^ | 3, 20, 41, 42 | Nonselected | Childhood | 0 | Group | Yes | Multisession | Active | Posttest only |
| Geraghty et al. (2010)^i^ | 37, 38, 48 | Nonselected | Middle adulthood | 95.62 | Individual | No | Multisession | Passive | Posttest only |
| Geraghty et al. (2010)^j^ | 1, 2, 37, 38 | Nonselected | Middle adulthood | 95.62 | Individual | No | Multisession | Passive | Posttest only |
| Grasso (2007) | 7, 8, 38 | Nonselected | Early adulthood | 89.50 | Individual | No | Multisession | Active | Short-term |
| Heinicke et al. (2007) | 1, 2, 3, 4, 17, 18, 19, 22, 26, 32, 34, 37, 38, 43, 44 | Nonselected | Adolescence | 100 | Group | Yes | Multisession | Passive | Posttest only |
| Jansen et al. (2008) | 4, 7 | Selected | Adolescence | 50 | Individual | Yes | Multisession | Passive | Posttest only |
| Lew et al. (2007) | 21, 23, 29, 35 | Selected | Early adulthood | 100 | Individual | Yes | Multisession | Active | Posttest only |
| Lindwall & Lindgren (2005) | 15, 20, 41, 42 | Nonselected | Adolescence | 100 | Group | Yes | Multisession | Passive | Posttest only |
| Martijn et al. (2012) - Study 2 | 35, 45 | Nonselected | Early adulthood | 100 | Individual | No | Single-session | Active | Posttest only |
| Martijn et al. (2010)^k^ | 45 | Selected | Early adulthood | 100 | Individual | No | Single-session | Active | Posttest only |
| Martijn et al. (2010)^l^ | 45 | Nonselected | Early adulthood | 100 | Individual | No | Single-session | Active | Posttest only |
| McCabe et al. (2006)^a, m^ | 3, 20, 27, 28, 29, 32, 33, 35, 42 | Nonselected | Childhood | 100 | Group | Yes | Multisession | Passive | Posttest only |
| McCabe et al. (2006)^a, n^ | 3, 20, 27, 28, 29, 32, 33, 35, 42 | Nonselected | Childhood | 100 | Group | Yes | Multisession | Passive | Posttest only |
| McCabe et al. (2006)^b, m^ | 3, 20, 27, 28, 29, 32, 33, 35, 42 | Nonselected | Childhood | 0 | Group | Yes | Multisession | Passive | Posttest only |
| McCabe et al. (2006)^b, n^ | 3, 20, 27, 28, 29, 32, 33, 35, 42 | Nonselected | Childhood | 0 | Group | Yes | Multisession | Passive | Posttest only |
| McLean et al. (2011) | 1, 2, 4, 7, 8, 11, 13, 17, 20, 21, 22, 24, 26, 31, 34, 35, 38, 39, 41, 42, 43, 48 | Selected | Middle adulthood | 100 | Group | Yes | Multisession | Passive | Posttest only |
| Murphy (1994)^k^ | 1, 2, 4, 6, 7, 9, 17, 18, 22, 29, 37, 38, 39, 40, 43, 46 | Selected | Early adulthood | 100 | Group | Yes | Multisession | Passive | Short-term |
| Murphy (1994)^l^ | 1, 2, 4, 6, 7, 9, 17, 18, 22, 29, 37, 38, 39, 40, 43, 46 | Nonselected | Early adulthood | 100 | Group | Yes | Multisession | Passive | Short-term |
| Özdemir et al. (2010)^o^ | 20 | Nonselected | Early adulthood | 0 | Group | Yes | Multisession | Passive | Posttest only |
| Özdemir et al. (2010)^p^ | 20 | Nonselected | Early adulthood | 0 | Group | Yes | Multisession | Passive | Posttest only |
| Özdemir et al. (2010)^q^ | 20 | Nonselected | Early adulthood | 0 | Group | Yes | Multisession | Passive | Posttest only |
| Paxton et al. (2007)^r^ | 1, 2, 3, 4, 17, 18, 22, 26, 32, 34, 37, 38, 43, 44 | Selected | Early adulthood | 100 | Group | Yes | Multisession | Passive | Posttest only |
| Paxton et al. (2007)^s^ | 1, 2, 3, 4, 17, 18, 22, 26, 32, 34, 37, 38, 43, 44 | Selected | Early adulthood | 100 | Group | Yes | Multisession | Passive | Posttest only |
| Pearson et al. (2012) | 1, 3, 7, 4, 6, 8, 10, 12, 13, 22, 38, 39, 40, 47, 48 | Selected | Middle adulthood | 100 | Group | Yes | Single-session | Passive | Short-term |
| Peterson et al. (2006)^t^ | 25, 46 | Nonselected | Early adulthood | 100 | Individual | No | Single-session | Passive | Posttest only |
| Peterson et al. (2006)^u^ | 21, 38, 39 | Nonselected | Early adulthood | 100 | Individual | No | Single-session | Passive | Posttest only |
| Ridolfi & Vander Wal (2008) | 21, 22, 25, 37, 39, 43 | Nonselected | Early adulthood | 100 | Group | Yes | Single-session | Active | Short-term |
| Rosen et al. (1995)^v^ | 1, 2, 3, 4, 7, 8, 10, 17, 18, 30, 37, 38, 39, 40 | Selected | Middle adulthood | 100 | Group | Yes | Multisession | Passive | Posttest only |
| Rosen et al. (1995)^w^ | 1, 2, 4, 6, 7, 9, 17, 18, 37, 38, 39, 40 | Selected | Middle adulthood | 100 | Group | Yes | Multisession | Passive | Posttest only |
| Rosen et al. (1989) | 1, 2, 7, 9, 17, 18, 37, 39, 40 | Selected | Early adulthood | 100 | Group | Yes | Multisession | Active | Short-term |
| Stanford & McCabe (2005) | 16, 21, 22, 31, 39 | Nonselected | Childhood | 0 | Group | Yes | Multisession | Passive | Posttest only |
| Waggoner (1999)^g^ | 1, 2, 4, 6, 9, 10, 17, 18, 37, 39, 40 | Nonselected | Childhood | 100 | Group | Yes | Multisession | Active | Posttest only |
| Waggoner (1999)^x^ | 1, 2, 4, 6, 9, 10, 17, 18, 37, 39, 40 | Nonselected | Childhood | 100 | Group | Yes | Multisession | Active | Posttest only |

^a^ Females. ^b^ Males. ^c^ Dance aerobics. ^d^ Step aerobics. ^e^ Video Intervention 1. ^f^ Video Intervention 2. ^g^ Cognitive-behavioural therapy (CBT). ^h^ Fitness training intervention. ^i^ Gratitude diaries. ^j^ Monitoring and restructuring. ^k^ High-risk women. ^l^ Low-risk women. ^m^ 3^rd^ and 4^th^ grade students. ^n^ 5^th^ and 6^th^ grade students. ^o^ Cycling. ^p^ Running. ^q^ Swimming. ^r^ Face-to-face intervention. ^s^ Internet intervention. ^t^ Feminist intervention. ^u^ Psychoeducation intervention. ^v^ Rosen, Orosan, & Reiter [76]. ^w^ Rosen, Reiter, & Orosan [77]. ^x^ Cognitive therapy. Gender is the percentage of female participants in the sample.

Change techniques are coded as follows: 1 = Discuss cognitions and their role in body image; 2 = Teach self-monitoring and restructuring of cognitions; 3 = Teach self-monitoring of behaviour; 4 = Change negative body language; 5 = Change the biased focus toward the body; 6 = Conduct guided imagery exercises; 7 = Conduct exposure exercises; 8 = Write about the body; 9 = Provide size-estimate exercises; 10 = Prompt action-planning; 11 = Teach time management skills; 12 = Agree on a contract; 13 = Barrier identification; 14 = Provide performance feedback; 15 = Provide encouragement; 16 = Prompt identification as a role model; 17 = Provide relapse-prevention strategies; 18 = Provide stress-management training; 19 = Provide alternative help resources; 20 = Provide physical activity exercises; 21 = Provide media literacy training; 22 = Discuss the beauty ideal; 23 = Teach strategies for resisting the effect of the media; 24 = Provide media-critique exercises; 25 = Provide alternative images of women and/or men; 26 = Discuss self-esteem; 27 = Provide self-esteem enhancement exercises; 28 = Discuss individual differences; 29 = Discuss alternatives to focusing on appearance; 30 = Discuss stereotypes; 31 = Discuss age-related issues and challenges; 32 = Discuss interpersonal relations; 33 = Teach interpersonal skills; 34 = Discuss social comparisons; 35 = Provide social comparison exercises; 36 = Provide a positive role-model; 37 = Discuss the concept of body image; 38 = Discuss the causes of negative body image; 39 = Discuss the consequences of negative body image; 40 = Discuss the behavioural expression of negative body image; 41 = Discuss healthy eating; 42 = Discuss physical activity; 43 = Discuss eating pathology; 44 = Discuss stress; 45 = Use evaluative conditioning; 46 = Discuss feminism; 47 = Discuss mindfulness; 48 = Provide mindfulness exercises; 49 = Technique not covered by the prior codes (specify).
